# Phase Segregation Mechanisms in Mixed-Halide CsPb(Br_*x*_I_1–*x*_)_3_ Nanocrystals in Dependence of Their Sizes and Their Initial [Br]:[I] Ratios

**DOI:** 10.1021/acsmaterialsau.3c00056

**Published:** 2023-09-06

**Authors:** Hannah Funk, Tal Binyamin, Lioz Etgar, Oleksandra Shargaieva, Thomas Unold, Alberto Eljarrat, Christoph T. Koch, Daniel Abou-Ras

**Affiliations:** † Helmholtz-Zentrum Berlin für Materialien und Energie GmbH, Hahn-Meitner-Platz 1, 14109 Berlin, Germany; ‡ The Institute of Chemistry, The Center for Nanoscience and Nanotechnology, The Casali Center for Applied Chemistry, The Hebrew University of Jerusalem, Jerusalem 9190401, Israel; § Humboldt-Universität zu Berlin, Institut für Physik, Newtonstraße 15, 12489 Berlin, Germany

**Keywords:** mixed-halide perovskite nanocrystals, transmission electron microscopy, phase segregation, in situ monitoring, multivariate analysis

## Abstract

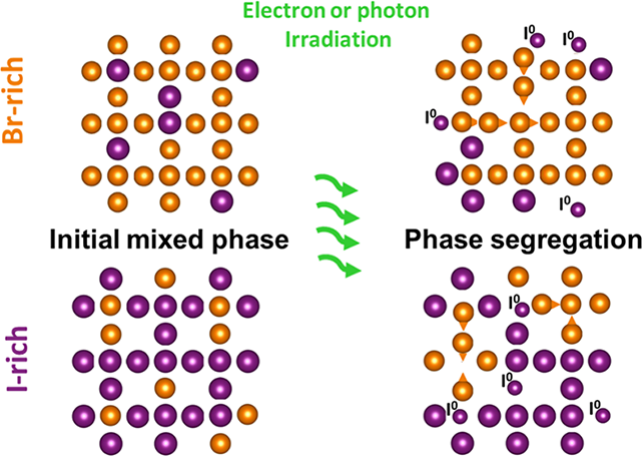

Phase segregation in inorganic CsPb(Br_*x*_I_1–*x*_)_3_ nanoparticles (NPs) exhibiting originally a homogeneous [Br]:[I] mixture was investigated by means of in situ transmission electron microscopy (TEM) and evaluated by using multivariate analyses. The colloidal synthesis of the NPs offers good control of the halide ratios on the nanoscale. The spatially resolved TEM investigations were correlated with integral photoluminescence measurements. By this approach, the halide-segregation processes and their spatial distributions can be described as being governed by the interaction of three partial processes: electron- and photon-irradiation-induced iodide oxidation, local differences in band gap energy, and intrinsic lattice strain. Since the oxidation can be induced by both electron-beam and light irradiation, both irradiation types can induce phase segregation in CsPb(Br_*x*_I_1–*x*_)_3_ compounds. This makes in situ TEM a valuable tool to monitor phase transformation in corresponding NPs and thin films on the sub-nm scale.

## 1 Introduction

Inorganic lead-halide-perovskite (LHP) with the stoichiometry A^+^B^2+^X^–^_3_ (A^+^ = formamidinium (FA^+^), methylammonium (MA^+^), Cs^+^; B^2+^ = Pb^2+^, Sn^2+^; X^–^ = Cl^–^, Br^–^, I^–^) exhibits an exceptional combination of optoelectronic properties: a sharp, optical absorption onset, low, nonradiative recombination rates, and high open-circuit voltages.^1^ Furthermore, the band gap energy is tunable over a range from 1.15 to 3.06 eV by interchanging and mixing A^+^ cations, B^2+^, and X^–^ halides.^2^ The possibility to design semiconductors with band gap energies of about 1.8 to 2.0 eV makes LHPs very suitable as functional layers in light-emitting diodes (LEDs) and as top cell absorbers in tandem solar cells. The perovskite absorber layer in such tandem devices can be composed of, e.g., Cs_0.05_(FA_0.77_MA_0.23_)_0.95_Pb(I_0.77_Br_0.23_)_3_, mixing the organic compounds FA^+^ and MA^+^ with the inorganic ions Cs^+^ and Pb^2+^ and the halides I*^–^* and Br*^–^*. Such tandem devices have reached power conversion efficiencies of above 33%.^3,4^

Nevertheless, the available band gap energies are limited by photoinduced halide phase segregation, a widely reported phenomenon that is not entirely understood that is attributed to be the cause for voltage losses in corresponding solar cells.^5−8^ Various models have been discussed for the redistribution of the mobile halides focusing on the influence of (i) differences in band gap energy,^9,10^ (ii) polaron-induced lattice strain,^11,12^ (iii) carrier gradients,^13^ or (iv) lattice strain due to different bonding lengths.^14,15^ All models agree in terms of that under equilibrium conditions, the entropic preference of APbX_3_ is halide mixing and that the process is reversible as long as the system is enclosed.^9,11,13^

Conclusively, more than one of these possible causes may be relevant at the same time. Instead of dividing the different aspects, we can classify them as processes driven by the minimization of the Gibbs free energy. Again, others observe a dynamic staged segregation.^16,17^ Illuminating the system deposits energy into it, which, in turn, may cause phase segregation. The phase segregation is determined by how much the illumination changes the kinetic and thermodynamic properties of the material.^18^

Furthermore, the loss of iodide, usually in the form of I_2_ or I_3_^–^, induced by light or chemical hole injection has been widely reported for mixed bromide/iodide perovskites that are not enclosed (for example nanoparticles, NP, or thin films in contact with a solvent).^19−21^ Recent publications describe halide oxidation and lead reduction (redox: 2I^–^ + Pb^2+^ → I_2_ + Pb^0^) induced by charge carriers as the underling process for halide segregation.^22−26^ Other authors refer to it more cautiously as the consequence of hole localization that neutralizes iodide locally.^27^ As long as the iodide is still in the system, it is possible for these redox reactions to occur in the opposite direction, which is why the effect is reversible in some cases.

The main method used in the present work to investigate LHPs is transmission electron microscopy (TEM). Electron microscopy is an appropriate tool to obtain and correlate spatially resolved information on morphology, structure, chemical composition, or even band gap energies. However, LHPs and especially organic LHPs are very electron-beam sensitive.^28^ The present work will therefore focus on the less sensitive inorganic cesium lead-halide perovskites. Also, for CsPbX_3_, it is crucial to know about the effects of the electron-beam irradiation to correctly characterize the material. Generally, an electron beam can cause (a) atomic displacement (knock-on damage), (b) radiolysis (ionization of material), i.e., losses between a few eV for excitation of conduction or valence electrons and tens or hundreds of eV for ionization of inner atomic shells, (c) creation of excitons (band transitions in the material), and (d) generation of phonons/heating in the investigated material. Detailed theory of the different interaction processes and their scattering cross-sections can be found in publications by Egerton et al.^29−34^ Dang et al. investigated experimentally the electron-beam damage in LHPs in detail^35−37^ and applied Egerton’s interaction theory to halide perovskites.^38^ From Dang’s work and also from the work by other authors,^39−41^ it has been established that radiolysis is the main damage process for electron irradiation in LHP. Since radiolysis is dominant at low electron-beam voltages,^42^ higher electron beam voltages are less harmful to LHPs.^36,41^ Furthermore, it is rather the total electron dose that is critical compared to the dose rate.^43^ For organic as well as for inorganic LHPs, the decomposition into PbX_2_ (X = I, Br, Cl) and metallic Pb has been reported by various authors.^36,40,44−47^ Due to the similar atomic interplanar distances of APbX_3_, PbX_2_, and Pb, decomposition products are often wrongly identified as perovskites in the literature.^48,49^

We would like to note that although energy-dispersive X-ray or electron energy-loss spectroscopy are established techniques for the study of elemental distributions in NPs on the (sub-)nm scale, these methods have never been successfully applied to the in situ monitoring of phase segregation in mixed-halide perovskite NPs. Therefore, only results from TEM imaging are shown and evaluated in the present work. Moreover, the presented TEM approach is not a high-throughput method, i.e., only a few nanoparticles can be investigated effectively. This fact contrasts with photoluminescence (PL) spectroscopy, which has been applied broadly to investigate phase segregation in nanocrystals. While these investigations are scientifically sound, the authors lacked the means to detect spatial phase distributions in nanocrystals due to the absence of high-resolution microscopy in their methods.

The present work reports about halide phase segregation in colloidal nanocrystals of mixed-halide CsPb(Br_*x*_I_1–*x*_)_3_ in dependence on their sizes and initial halide ratios [Br]:[I] and provides further confirmation that halide oxidation indeed plays a key role in halide segregation. The phase segregation was induced and monitored in situ by high-resolution TEM (HRTEM) on the sub-nm scale and evaluated by multivariate analysis (MA). Furthermore, the results were correlated with light-induced phase segregation monitored by PL spectroscopy. For the discussion, the findings of the present work were compared with the findings of a previous study by Funk et al.^50,51^

## 2 Experimental Methods

### 2.1 Sample Synthesis

Chemicals: cesium carbonate (Cs_2_CO_3_, 99.9%, Sigma-Aldrich), lead(II) bromide (PbBr_2_, ≥98%, Sigma-Aldrich), lead(II) iodide (PbI_2_ 99.99%, Sigma-Aldrich), oleic acid (OA, 90%, Sigma-Aldrich), oleylamine (OLAM, 70%, Sigma-Aldrich), 1-octadecene (ODE, 90%, Sigma-Aldrich), ethyl acetate (≥99.5%, Sigma-Aldrich), and hexane (technical grade, Bio-lab) were used as received, without any further purification.

#### 2.1.1 Step 1: Preparation of Cs-Oleate

The Cs-oleate precursor was prepared according to a previously published procedure by Protesescu et al.^52^ and by us. In a 100 mL 3-neck flask, 0.6 mmol (0.2 g) of Cs_2_CO_3_ was mixed with 625 μL of oleic acid (OA) and 7.5 mL of 1-octadecene (ODE). The solution was degassed for 1 h under vacuum conditions at 120 °C and then heated up to 150 °C under Ar flow.

#### 2.1.2 Step 2: Synthesis of CsPb(Br_*x*_I_1–*x*_)_3_ (X = Br, I) NPs

The NPs were synthesized according to a previous report.^52^ First, different ratios of PbBr_2_ and PbI_2_ (for *x* = 0.8, 0.4) to a total of 0.188 mmol (0.0552 g of PbBr_2_ and 0.0172 g of PbI_2_ for *x* = 0.2) were mixed with 0.3 mL of OA, 0.3 mL of OLA, and 5 mL of ODE in an additional 100 mL 3-neck flask. The solution was degassed for 1 h under vacuum at 120 °C and then heated up to 150 °C for small NPs and up to 170 °C for bigger NPs under Ar flow. The reaction was carried out by injecting 0.4 mL of the Cs-oleate precursor solution into the PbX_2_ precursor solution. The reaction was quenched using an ice bath after a few seconds. Ethyl acetate was added to the crude solution in a volume ratio of 3:1, and the NPs were centrifuged at 6000 rpm for 10 min. The precipitate was dispersed in hexane for further characterization.

### 2.2 Structural Characterization

Time series in transmission electron microscopy: TEM imaging was performed using a JEOL JEM2200FS and a 1k slow-scan charge-coupled device (CCD) camera (MSC, Gatan Inc.) at room temperature using electron dose rates given in Table S6 and an accelerating voltage of 200 kV. The electron beam was not blanked at any instant during each time series. The magnification was calibrated using a Si wafer. It should be noted that reversibility of the phase-segregation process cannot be investigated by TEM analyses since further transformations happening in the electron beam right after the phase-segregation process result in the irreversible destruction of the nanoparticles.

### 2.3 Multivariate Analysis (MA)

Evaluation of TEM time series with multivariate analysis: The two methods used to evaluate a stack of high-resolution TEM images are principal component analysis (PCA) followed by an independent component analysis (ICA).^53,54^ While PCA uses a reduction of the dimensionality to simplify the problem at hand, ICA postulates the independence of the source variables. ICA is a special case of a blind source separation and is widely used in digital image processing. For more details on this approach, the reader is referred to refs (50) and (55).

### 2.4 Photoluminescence Measurements

PL spectra for drop-cast NPs with side lengths of 20–100 nm were acquired by a 1/2 m grating monochromator coupled with a CCD detector. The investigated samples were excited by means of a diode laser emitting light at a wavelength of λ = 409 nm (a spot radius of approx. 100 μm and excitation intensity in the range of 0.3–1.9 W cm^–2^). The same conditions were used for the drop-cast NPs with side lengths of 20–100 nm. PL spectra of the NPs with a side length of 10–22 nm in solution were measured using an optical flame spectrometer from Ocean Optics and excited by means of an ultraviolet-LED lamp (λ_max_ = 365 nm) at an intensity of about 1 sun (1 kW/m^2^) in a N_2_ atmosphere. The excitation light was cut band limited using a short-pass filter at 500 nm.

## 3 Results

### 3.1 Size Dependence of Phase Segregation in Mixed-Halide NPs

NPs of the composition CsPb(Br_0.4_I_0.6_)_3_ and CsPb(Br_0.8_I_0.2_)_3_ were synthesized by colloidal synthesis. This process ensures a better control of the iodide/bromide ratio on all scales compared with spin-coated samples and allows for comparability of the results obtained by HRTEM and by integral PL spectroscopy.

**Figure 1:**
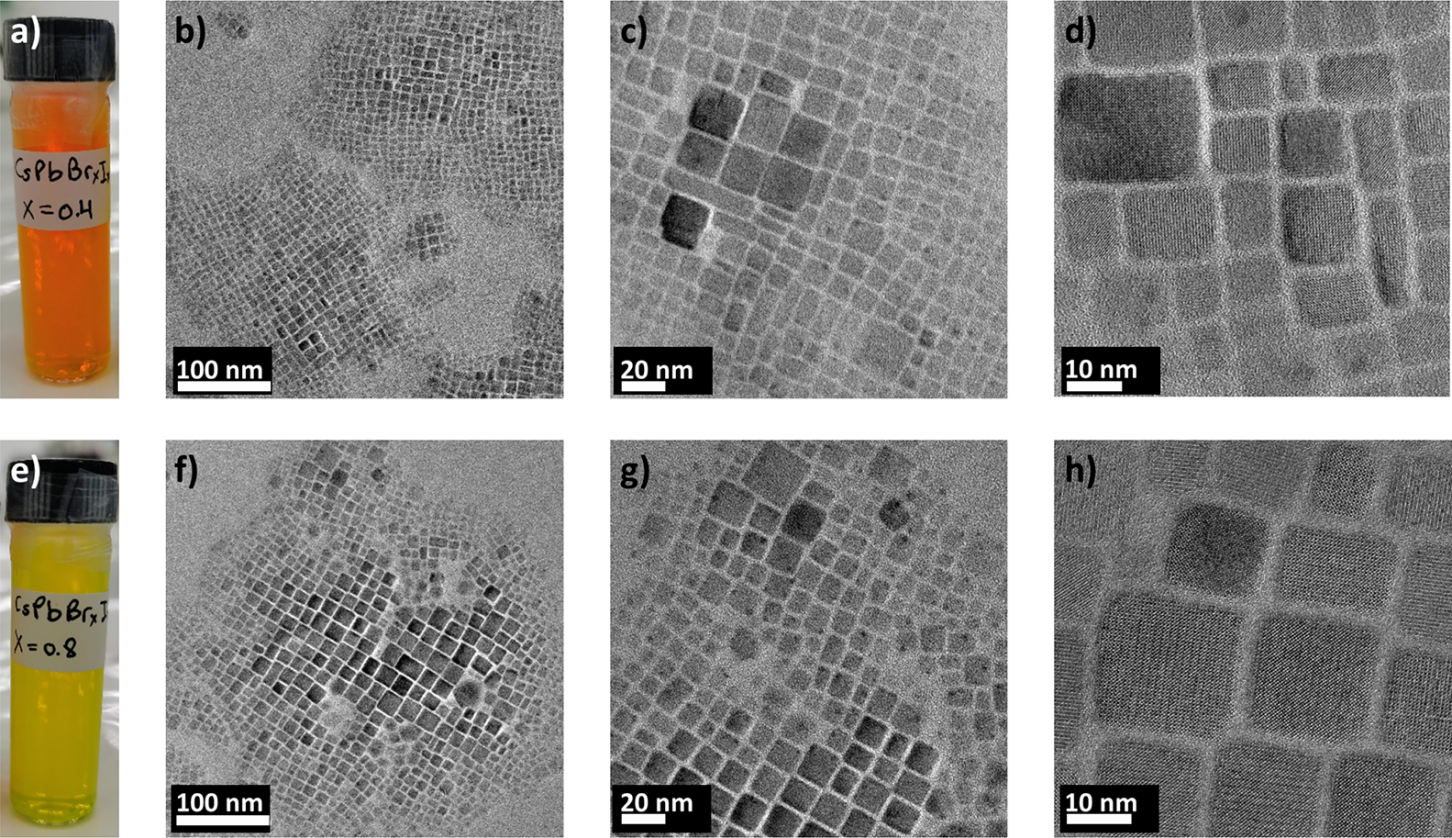
(a) Iodide-rich CsPb(Br_0.4_I_0.6_)_3_ NPs (orange) and (e) bromide-rich CsPb(Br_0.8_I_0.2_)_3_ NPs (yellow) dispersed in hexane. The tunability of the band gap energy is highlighted by the different colors of these solutions. (b–d) TEM images acquired at various magnifications showing CsPb(Br_0.4_I_0.6_)_3_ NPs as well as CsPb(Br_0.8_I_0.2_)_3_ NPs in (f–h). The particle side lengths vary between about 10 and 22 nm.

Figure 1a,e shows the NPs dispersed in hexane. They exhibit the typical bright orange and yellow colors for iodide-rich and bromide-rich CsPb(Br_*x*_I_1–*x*_)_3_, visualizing the tunability of the band gap energy via the [Br]:[I] ratio. The band gap energies were determined to be around 2.23 (556 nm) for CsPb(Br_0.4_I_0.6_)_3_ and 2.38 eV (520 nm) for CsPb(Br_0.8_I_0.2_)_3_ by PL and absorption spectroscopy as depicted in Figure S1. These values are in good agreement with those reported in the literature^7,21,52,56−59^ given in Table S1.

The NPs were drop-cast on a carbon-coated TEM grid, where they assembled in clusters. The larger particles are usually found in the center of a cluster. They are mostly of cubic shape and vary in side length between 10 and 22 nm for both the iodide-rich and the bromide-rich NPs (see Figure 1b–d,f–h).

#### 3.1.1 In Situ TEM Results

**Figure 2:**
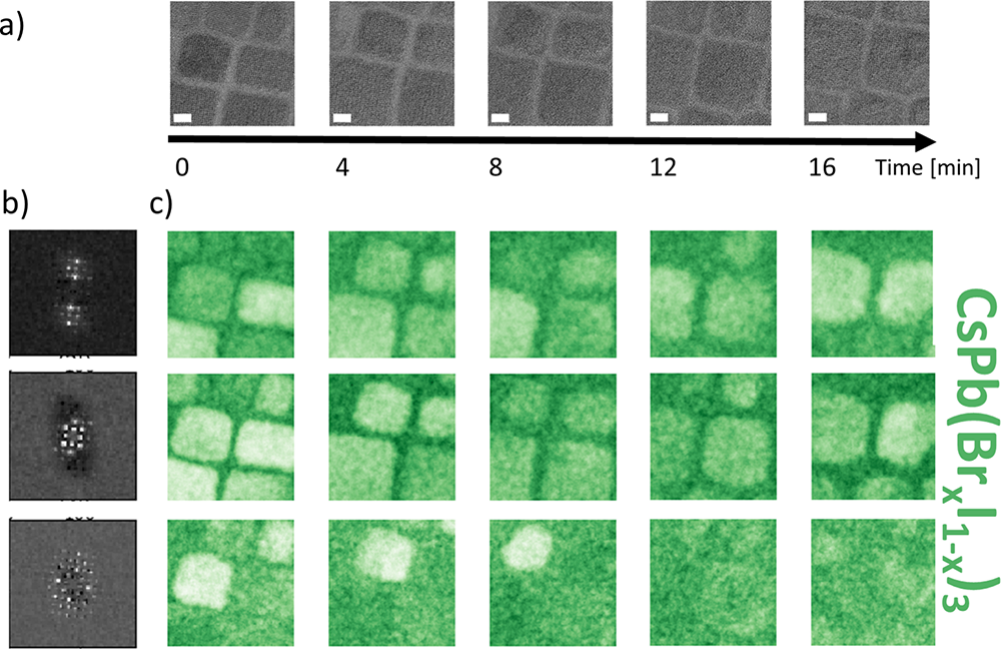
(a) HRTEM images acquired during the 17 min of total acquisition duration (each image 4 min); (b) characteristic diffraction patterns of the several differently orientated structures assigned to CsPb(Br_0.8_I_0.2_)_3_; (c) corresponding abundance maps showing no halide phase segregation, but a slow amorphization. High intensity in a pixel corresponds to high abundance of the corresponding diffractogram in that pixel. The scale bar has a length of 5 nm.

HRTEM time series were acquired over a duration of up to 20 min on iodide-rich CsPb(Br_0.4_I_0.6_)_3_ and bromide-rich CsPb(Br_0.8_I_0.2_)_3_ NPs to monitor electron-beam-induced phase segregation. No such phase segregation was detected for the NPs in the various time series. Figure 2a shows the HRTEM time series of CsPb(Br_0.8_I_0.2_)_3_ NPs over a period of 17 min. Slight morphological changes are visible. A multivariate algorithm^50,55^ was used to identify and map the structural information from the local atomic structure. Several diffraction patterns were acquired during the time series (Figure 2b); all of these patterns were assigned successfully to the CsPb(Br_0.8_I_0.2_)_3_ phase by determining the interplanar distances from the reflection positions. The different symmetries of the diffraction patterns can be explained by slightly different crystal orientations of the NPs with respect to the incident electron beam.

In the abundance maps in Figure 2c, a high intensity corresponds to a high abundance of the structure in this pixel. While for phase segregation new phases should appear over time, this is not the case for the small NPs. No new diffraction patterns indicating the evolution of phase segregation or the formation of another phase appear during the time series. The only change observed is that the initial patterns vanish, indicating amorphization. During this process, neither halide phase segregation nor the formation of lead-halide particles was observed. Also, in iodide-rich CsPb(Br_0.4_I_0.6_)_3_ NPs, no phase segregation was detected.

In spite of the higher magnification and a higher electron dose, the present NPs are much more stable under the electron beam compared to the bigger crystallites of about 40 nm × 40 nm size presented in Funk et al.^50^ No phase segregation induced by electron-beam irradiation was observed for iodide-rich CsPb(Br_0.4_I_0.6_)_3_ or bromide-rich CsPb(Br_0.8_I_0.2_)_3_ NPs of 10–22 nm side lengths. This result is in good agreement with previous PL measurements on CsPb(Br_*x*_I_1–*x*_)_3_ NPs that show the absence of phase segregation below a certain NP size.^9,10,21^ In the next subsection PL measurements will be correlated with the TEM results and discussed with respect to results in the literature.

#### 3.1.2 In Situ PL Results

**Figure 3:**
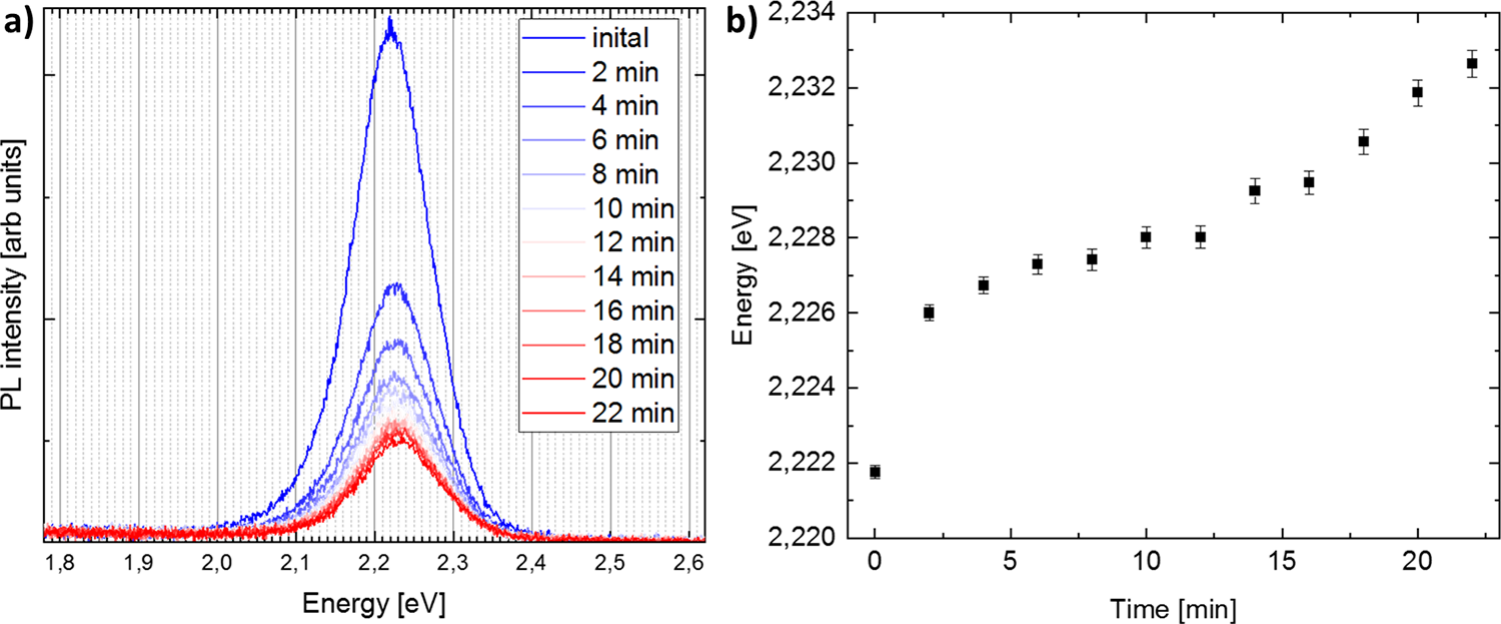
(a) PL intensity of CsPb(Br_0.8_I_0.2_)_3_ NPs excited with a white LED lamp over a time period of 22 min. (b) Lorentzian fit of PL peak energy indicating a blue shift of 11 meV (2.7 nm).

Figure 3a shows the PL spectra excited by means of a white LED lamp over a time period of about 22 min. The PL peak does not exhibit a red shift, reported as an indication for halide segregation in bulk material,^5,7,8,13,60^ but a blue shift of a few nm was detected for mixed-halide NPs.^9,21^ The band gap energy of initially 2.22 eV (559 nm) is in good agreement with the literature and deviates slightly from the value given in Figure S1. This is probably due to another NP concentration in the solution during the measurement since the hexane evaporated during storage and had to be filled up from time to time.

Figure 3b shows the Lorentzian fit of the PL signal in Figure 3a and displays a blue shift of 2.7 nm for 22 min of illumination in an inert atmosphere. A filter effect caused by reabsorption makes the higher energy leg steeper.^61^ Therefore, the peak is not symmetric and not strictly Lorentzian, hence, making the fitting error potentially larger than indicated in Figure 3b. However, the tendency is clear and in-line with the literature. Draguta et al.^9^ described a 3 nm blue shift after 120 s of excitation (405 nm, *I*_exe_ = 60 mW cm^*–*2^) in CsPb(Br_0.5_I_0.5_)_3_. Zhang et al.^21^ described an intensity-dependent blue shift in CsPb(Br_0.4_I_0.6_)_3_ of 17 nm after 10 min (405 nm, *I*_exe_ = 30 mW cm^*–*2^) and of 115 nm after 80 min (405 nm, *I*_exe_ = 15 mW cm^*–*2^). Furthermore, their measured blue shift is reversible in ensemble films consisting of packed NPs but not reversible in individual NP.^21^ The smaller shift in comparison with literature reports detected in the present work may be explained either by the lower intensity of the LED lamp used in the present work or by the inert atmosphere in which the PL measurement was performed.

Both the polaron-based model for halide-phase segregation by Bischak et al.^12^ and the band gap-based model by Draguta et al.^9^ predict an NP size threshold below which no phase segregation occurs. The polaron-based model rationalizes irradiation-induced lattice vibrations as a possible cause for the halide redistribution. It predicts a minimum size of 8 nm × 8 nm for phase segregation to occur. However, the present work and reports in the literature^9,10^ show that phase segregation does not take place in particles with edge lengths of more than 20 nm × 20 nm. In the second model, the band gap differences between I-rich and Br-rich regions cause charge-carrier accumulation in I-rich domains, which again induces the iodide to diffuse to these regions to reduce the free energy. According to the model proposed by Draguta et al.,^9^ the segregation rate is dependent on the excitation intensity *I*_exc_ as well as on the carrier diffusion length *l*_e/h_. In the case that the particle is smaller than the diffusion length, no phase segregation can occur. This model is supported by the presented results and experiments performed by Gualdrón-Reyes et al.,^10^ who reported that, while a blue-shift was detected for NPs with a side length of less than about 46 nm, a red-shift was found for NPs with a side length greater than about 46 nm (405 nm, *I*_exe_ = 10 mW cm^*–*2^).

It can be concluded that the TEM results showing no phase segregation are in good agreement with the PL results and those reported in the literature. A shift of 2.7 nm in the PL peak, as observed by PL during 22 min of irradiation, corresponds to a change in lattice parameters that lies below the detection limit of the phase assignment by TEM.

### 3.2 Spatial Evolution of Phase Segregation in Iodide-Rich Nanoparticles with Side Lengths of >40 nm

As described in the previous subsection, halide segregation is not observable in TEM or PL for small (20–22 nm side length) mixed-halide NPs. However, electron-beam-induced phase segregation for a crystallite with a side length of about 40 nm was indeed observed as described in Funk et al.^50^ and photoinduced phase segregation for particles with a side length of 46 nm or larger was reported using PL analysis.^10^ These results are explained well by the band gap-based model,^9^ which predicts a size threshold below which light-induced phase segregation is not occurring. Hence, CsPb(Br_0.4_I_0.6_)_3_ NPs with a wider size distribution were produced by changing the temperature during step 2b of the colloidal synthesis.

**Figure 4:**
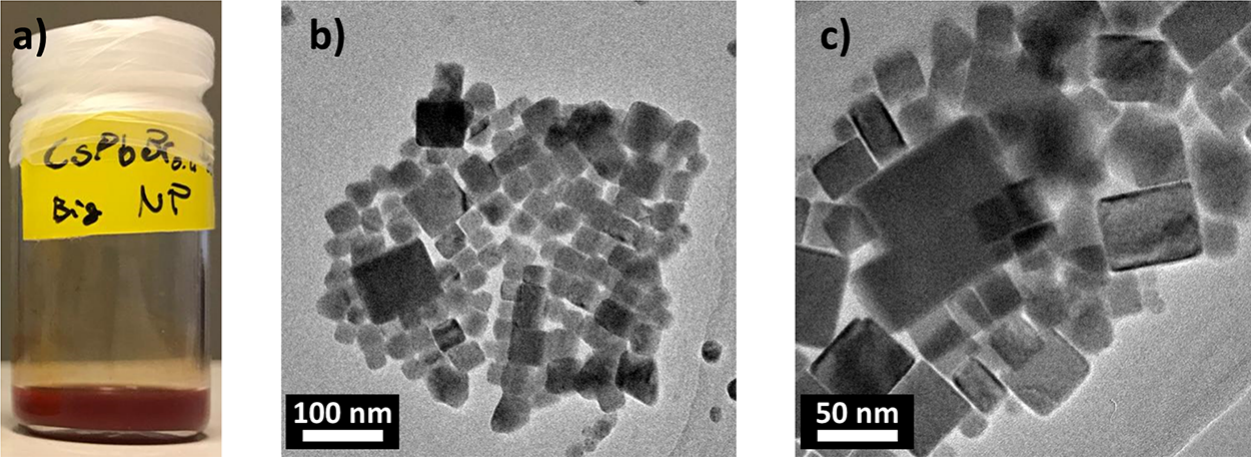
(a) Iodide-rich CsPb(Br_0.4_I_0.6_)_3_ NPs (dark red) dispersed in hexane. (b, c) TEM overview images of CsPb(Br_0.4_I_0.6_)_3_ NPs with side lengths from 20 to 100 nm.

Figure 4a shows the solution containing large, iodide-rich CsPb(Br_0.4_I_0.6_)_3_ NPs (dark red), in comparison with the small, bromide-rich CsPb(Br_0.8_I_0.2_)_3_ (yellow) and iodide-rich CsPb(Br_0.4_I_0.6_)_3_ (orange) NPs. As depicted in Figure 4b,c, the iodide-rich particles are mostly of cubic shape, and their side lengths range from about 20 to 100 nm.

#### 3.2.1 In Situ TEM Results

Probing particles with side lengths between 40 and 50 nm at a magnification of 300,000 proved to be the right tradeoff between a high resolution and more or less full view of one crystallite sufficiently large to monitor the halide phase segregation. In the following, halide phase segregation is induced and monitored at two different positions of the same sample, which will be referred to as position A and position B.

#### 3.2.2 Multivariate Analysis

**Figure 5:**
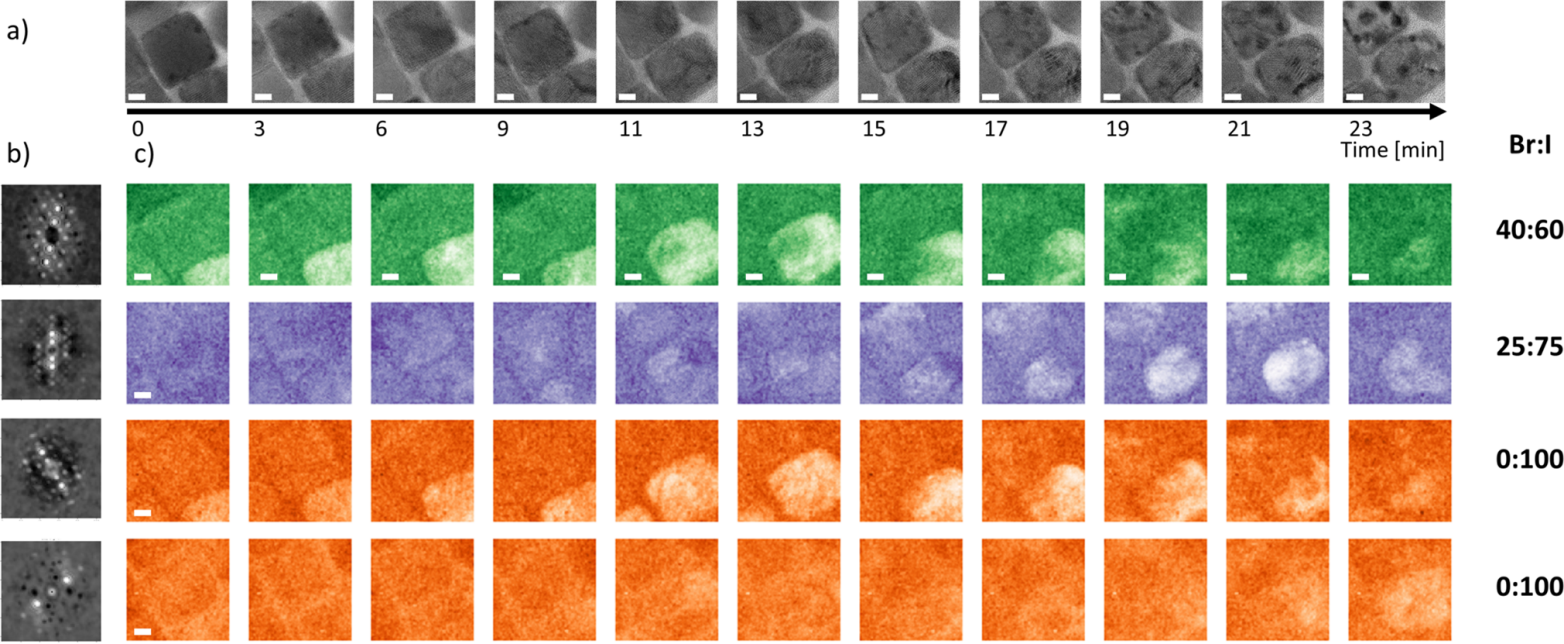
Multivariate analysis of a 40 nm × 45 nm large CsPb(Br_0.4_I_0.6_)_3_ crystallite at position A. (a) HRTEM images acquired during 23 min of total acquisition duration, (b) characteristic diffraction patterns of most abundant structures of the upper crystallite, (c) corresponding abundance maps identified as CsPb(Br_*x*_I_1–*x*_)_3_ for *x* = 0.4, 0.25 and 0 (green, purple, and orange). As in Figure 3, a high intensity in a pixel corresponds with a high abundance of the structure in this pixel. The length of the scale bars is 10 nm.

To analyze the evolution of the electron beam-induced phase segregation in CsPb(Br_0.4_I_0.6_)_3_ NPs, multivariate statistical analysis (MSA) was applied to HRTEM time series acquired at different positions of the sample. Figure 5a shows a HRTEM time series recorded at position A over a time period of 23 min. As in the previous section, the algorithm virtually scanned each HRTEM image, collecting the structural information encoded in the FFTs of small patches of the atomic-resolution images. Then, the above mentioned PCA & ICA methods were used to reduce the dimensionality of the data set and to identify the abundant crystalline phases in form of characteristic diffractograms. Figure 5b displays the most abundant components of the diffractograms for the lower particle in the HRTEM images. The abundance maps in Figure 5c display the evolution of the corresponding structure over time. A high contrast pixel in the abundance maps corresponds to a high abundance of the respective structure in this pixel.

The abundance maps in Figure 5c show how the lower particle of initially CsPb(Br_0.4_I_0.6_)_3_ (green) transforms into an iodide-richer region of CsPb(Br_0.25_I_0.75_)_3_ (purple) as well as into pure CsPbI_3_ (orange, in two orientations) during the first 12 min, before additional further transformations occur under the electron beam, and the particle decomposes as visible in the HRTEM images. The upper particle in Figure 5 exhibits a very similar segregation behavior as well as the evolution of Pb during the later stages when the particle decomposes visibly but is oriented differently. Its multivariate analysis can be found in Figure S3, and HRTEM results can be found in Figure S4.

**Figure 6:**
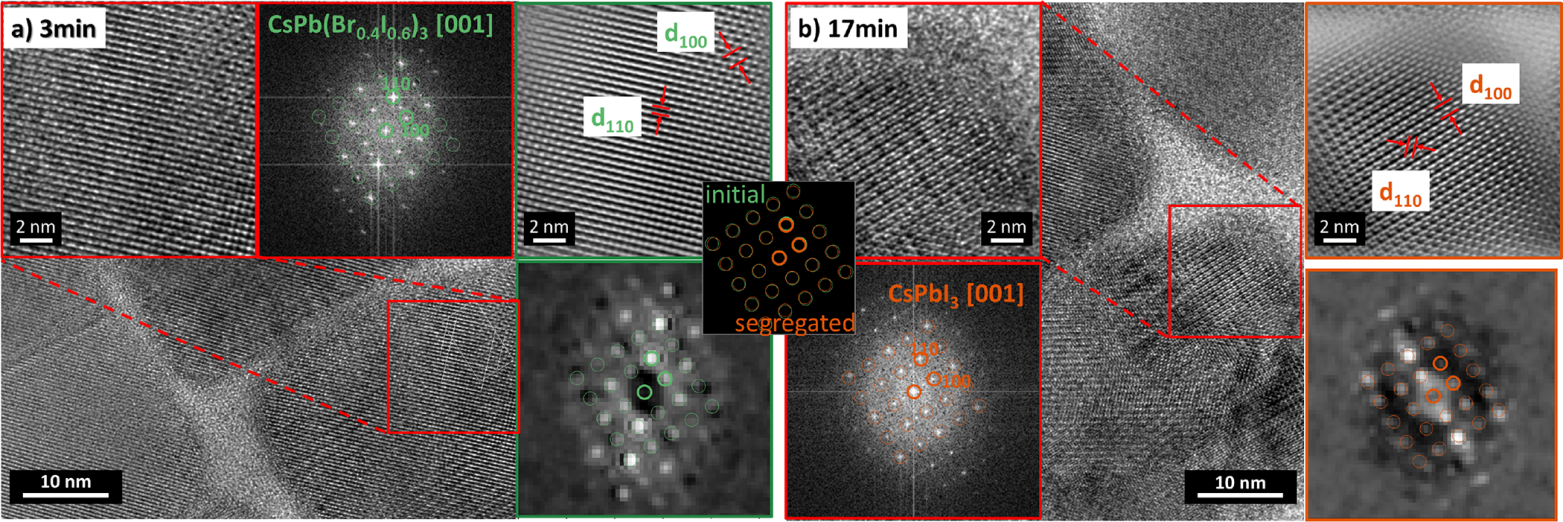
Phases assignment at position A: (a) HRTEM after 3 min and (b) 17 min of electron beam irradiation. For exact phase assignment, the FFT of the image detail (red border) matching the diffraction pattern determined by the MSA algorithm is chosen. The exact interplanar distances are obtained from the Fourier filtered image, for initial CsPb(Br_0.4_I_0.6_)_3_ (green) and terminal CsPbI_3_ (orange). Please note that the zone axes of the crystallites are not perfectly aligned parallel to the incident electron beam.

The [Br]:[I] ratio of each diffraction pattern was identified by using Vegard’s law^62^ as well as the cubic lattice parameters of 5.874 Å for CsPbBr_3_^63^ and 6.289 Å for CsPbI_3_,^64^ since the deviations from the actual orthorhombic phase of CsPbX_3_ NPs at room temperature lie below the detection limit of HRTEM.^65^ To obtain a higher resolution, the interplanar distances were determined from the original images, instead of from the more pixelated diffractograms. Figure 6 shows exemplarily how, for each abundant structure, the FFTs generated by the MSA algorithm (green/orange border) were compared with the FFTs of the original image (red border) of a region with a high abundance of the corresponding structure. Then, the interplanar distances were measured in the Fourier filtered HRTEM images (green/orange border). A deviation of about ±5% from the halide ratio results from the spatial resolution and inaccuracy of the image extraction. Hence, the resulting phase assignment is to be understood as a trend of the spatial evolution of phase segregation.

**Figure 7:**
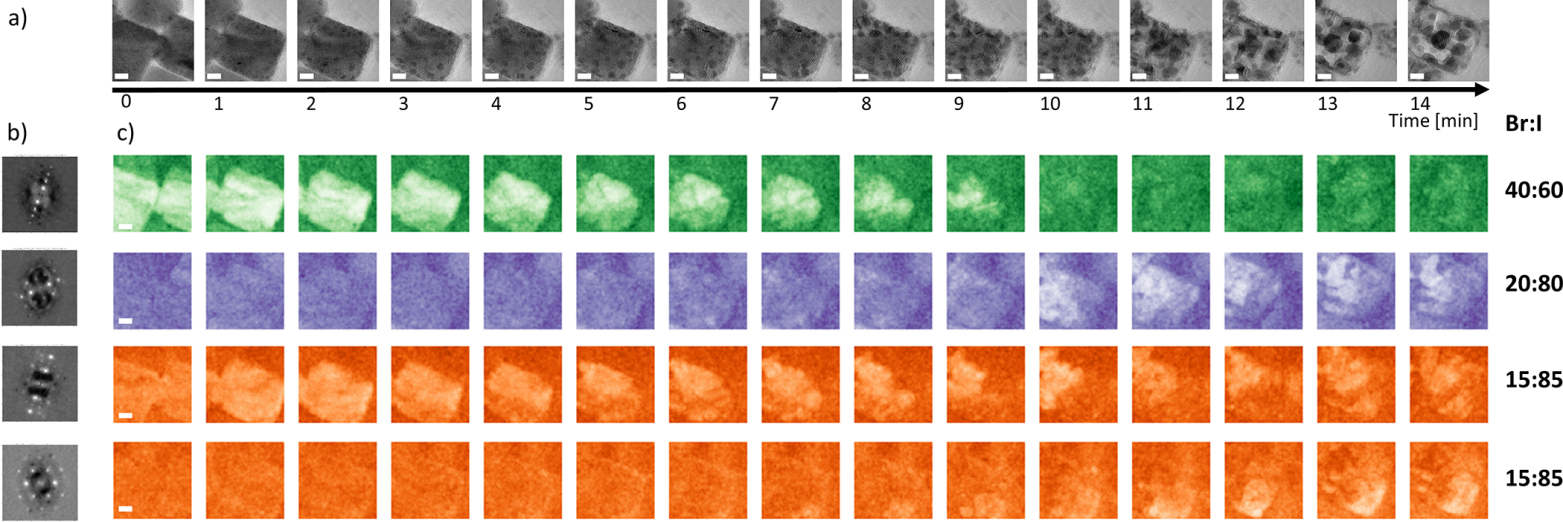
Time series of an initially 40 nm × 50 nm large CsPb(Br_0.4_I_0.6_)_3_ crystallite at position B. (a) HRTEM images acquired during 14 min of total acquisition duration; (b) characteristic diffraction patterns of the most abundant structures as identified by multivariate analysis; (c) corresponding abundance maps identified as CsPb(Br_*x*_I_1–*x*_)_3_ for *x* = 0.4, 0.20, and 0.15 (green, purple, and orange). High intensity in a pixel corresponds to a high abundance of the structure in this pixel. Scale bars are 10 nm.

Figure 7 shows a second HRTEM time series and its multivariate analysis at position B on the same TEM grid. While the trend of the phase segregation and the following further transformations leading to decomposition are the same as at position A, both processes happen much faster. After about 5 min, the initial CsPb(Br_0.4_I_0.6_)_3_ (green) compound transforms into iodide-richer regions of CsPb(Br_0.2_I_0.8_)_3_ (purple) and CsPb(Br_0.15_I_0.85_)_3_ (orange). At the same time, high-contrast particles are much more visible at position B, indicating the presence of bromide-rich areas that are not resolved by the multivariate analysis.

#### 3.2.3 Bromide Inclusions

**Figure 8:**
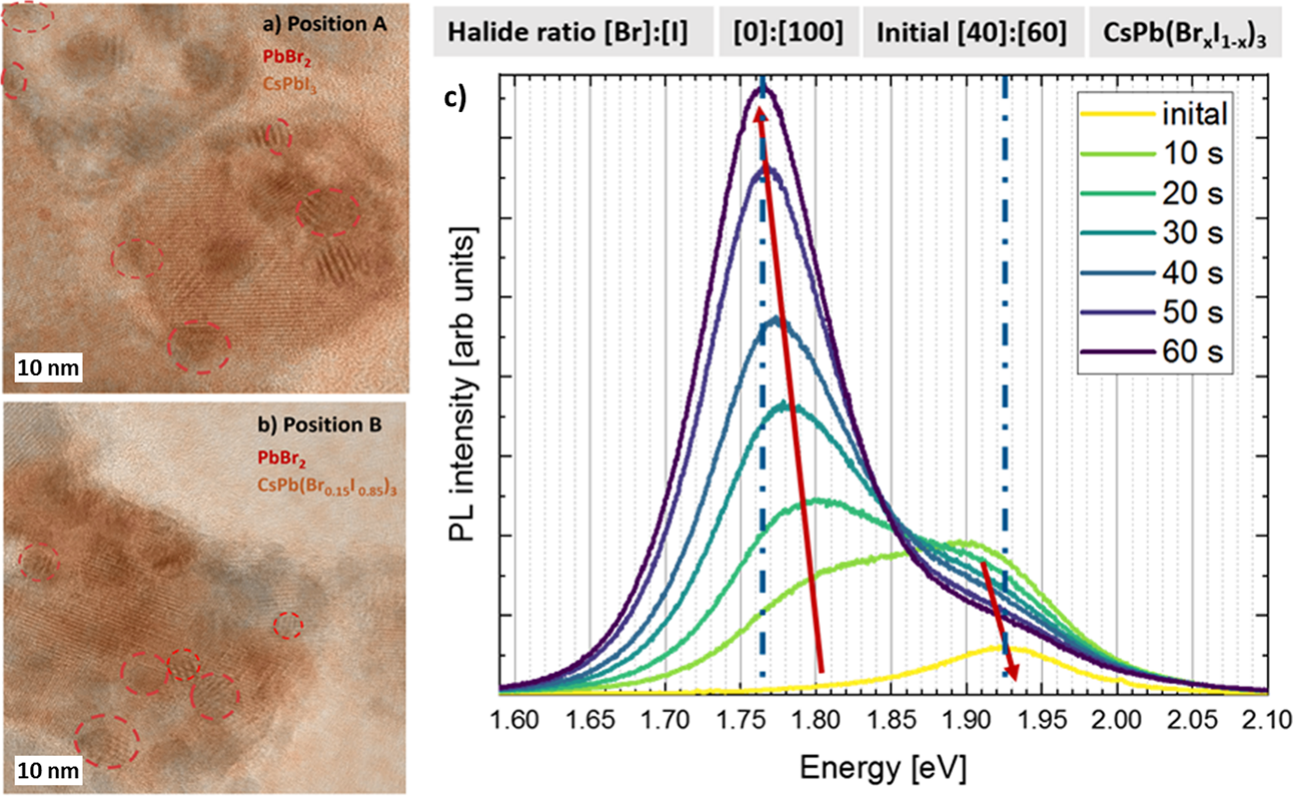
HRTEM image with the overlay of the I-rich ICA abundance map for (a) position A after 23 min and (b) position B after 10 min of electron-beam irradiation. In both time series, inclusions of Br-rich areas are present that can be identified as PbBr_2_. (c) PL peak intensity of initial CsPb(Br_0.4_I_0.6_)_3_ excited with a 409 nm diode laser measured over 1 min. The initial peak red shifts from 1.93 eV (645 nm) correspond to a [Br]:[I] ratio of [40]:[60] toward a stable value of about 1.77 eV (700 nm) corresponding to a ratio of [0]:[100].

As described in the last subsection, for both positions A and B, the initially I-rich CsPb(Br_0.4_I_0.6_)_3_ crystallites developed into pure or almost pure CsPbI_3_, while no region enriched in bromide was detected by means of PCA/ICA (see Figures 5 and 7). Detailed evaluation of the HRTEM by means of ROI-FFT analysis (see Figures S4 and S5) images shows that bromide-rich areas can be found as inclusions of up to 5 nm in size that are too small to be picked up by the MSA algorithm. Figure 8a,b shows these small bromide-rich inclusions at position A as well as at position B in the I-rich matrix.

Because of the structural similarity of the ternary, the binary, and the pure lead phases (e.g., CsPbBr_3_, PbBr_2_, Pb_0_), it is not always possible to identify one of these phases unambiguously by means of HRTEM. This is especially true in the case that only one direction of lattice planes is resolved in the TEM images. Due to the mass-thickness contrast effect, small high-contrast areas are usually attributed to PbBr_2_;^35,37,66^ this is probably also the case in the present work. Also, the differences in the interplanar distances are often smaller than the measurement error. A careful evaluation comparing FFTs with simulated electron diffraction patterns of the different phases in question was conducted using the JEMS software.^67^ Interplanar distances and phase assignments can be found in Section 1 of the Supporting Information.

#### 3.2.4 Time Scale, Intensity, and Residual Organic Material

It is noteworthy that the time scales of the processes in the time series described above differ substantially. While the halide phase segregation at position A (Figure 5) occurred during the first 12 min, at position B, the phase segregation evolved already during the first 5 min before further transformation started to dominate the process (Figure 7). This means that more than twice the total electron dose was necessary to induce phase segregation at position A, in comparison with the particle at position B. Other particles did not show any phase decomposition during exposure of up to 20 min, while at other positions, the decomposition was so fast under the identical conditions that halide-phase segregation was not possible to be analyzed before the particle decomposed (see Figure S6). As described in detail in Section 2 of the Supporting Information, this different behavior in the electron beam is not related to the shape of the NPs or to other properties of the corresponding crystallite (as, e.g., attached ligands) but dominated by local fluctuations of residual organic material from the solution in which the NPs were dispersed and drop-cast. The electron-beam irradiation can decompose hydrocarbons from the organic material and deposit carbon on the sample. Subsequently, further adsorbates diffuse from the surrounding area into the illuminated area. The carbon acts as a protective layer and impedes the volatilization of halide ions from the sample. The suppression of degradation of LHP by carbon coating has been reported in the literature.^47^ These effects vary with the thickness of the carbon layer and, hence, with the available organic material in the environment of the electron beam illuminated area.

In conclusion, the large, I-rich CsPb(Br_0.4_I_0.6_)_3_ NPs indeed exhibit electron-beam-induced phase segregation into a pure CsPbI_3_ matrix with Br-rich inclusions. The time scale and occurrence of this process in TEM are dependent on the amount of residual organic material in the specimen. This result indicates that the phase-segregation process is illumination-intensity dependent as predicted by the band gap-dependent phase-segregation models.^9,13,68^ In addition, it is important to be aware of the further transformations that occur under the electron beam after the segregation. These further transformations are facilitated by elevated temperatures.^36^ An estimate for electron-beam-induced heating is provided in Section 3 of the Supporting Information.

#### 3.2.5 In Situ PL Results

Figure 8c shows the PL measurement performed on large drop-cast NPs in air. The PL peak redshifts from an initial value of 1.93 eV (645 nm) corresponding to a [Br]:[I] ratio of about 40:60 toward a stable value of about 1.77 eV (700 nm) that can be identified as pure CsPbI_3_. The peak approximate halide ratio was identified with the literature values presented in Table S1.^5,7,8,13,52,59,60,69^

The red shift and the initial and final values of the PL measurements are in very good agreement with the in situ TEM measurements. Both TEM and PL results indicate that the initially I-rich [Br]:[I] ratio of 40:60 CsPb(Br_*x*_I_1–*x*_)_3_ crystallites segregated into pure CsPbI_3_ (and secondary phases observable by TEM). Even though the differences between light and electron-beam irradiation give rise to different time scales that make it difficult to quantitatively compare the intensities, the present work shows that halide phase segregation can be induced by both electron-beam or photon irradiation.

#### 3.2.6 Side Note on the Terminal [Br]:[I] Ratio for Halide Segregation

For LHP thin films, a terminal [Br]:[I] ratio for iodide-richer phases, below which no halide segregation occurs, is observed experimentally. CsPb(Br_*x*_I_1–*x*_)_3_ thin films with [Br]:[I] ratios of higher iodine content than [I]/([Br] + [I]) > 60% are reported to be stable against photoinduced halide-segregation.^7^ (For MAPb(Br_*x*_I_1–*x*_)_3_, stable ratios of [I]/([Br] + [I]) > 80% are reported.^5^) Brennan et al.^13^ gave an overview of the extensive literature on the halide-segregation threshold and proposed explanations. However, none of them can explain conclusively the A cation-independent threshold below which no halide segregation occurs.

In the present work, no [Br]:[I] threshold ratio for phase segregation was found in individual NPs of up to 100 nm. As predicted theoretically,^68^ a complete segregation, resulting in a pure CsPbI_3_ phase and a Br-richer phase, was observed. In light of the threshold-riddle in thin films, the present results are intriguing. Hutter et al. reported that the threshold value can be increased, i.e., more ratios can be stabilized against segregation by applying external physical pressure or chemically compressing the unit cell by integrating smaller Cs^+^ cations into a MAPb(Br_*x*_I_1–*x*_)_3_ lattice.^15^ The absence of neighboring grains and/or the volume-to-surface ratio, i.e., the size of the NPs, are likely to play a role for the absence of a threshold ratio reported in the present work and could serve as a starting point to solve the riddle in thin films to eventually stabilize more band gaps for optoelectronic applications.

## 4 Discussion

As discussed in detail in Section 3.1, it was shown that halide phase segregation in NPs smaller than 23 nm side length does not take place in TEM or PL as predicted by the band gap-based model^9^ for halide phase segregation. In accordance with the literature, the PL peak exhibits a slight blue shift, indicating a slow outgassing of iodide from small CsPb(Br_0.8_I_0.2_)_3_ NPs, corresponding to a change in the halide ratio that lies below the identification threshold of HRTEM phase assignment. The results of the present work show that the intensity-dependent size threshold of about 40 nm for photosegregation, as predicted by the band gap model, is also given for electron irradiation. Section 3.2 shows phase segregation initiated by an electron beam in TEM as well as light by PL on the same sample. For both methods, the initially I-rich CsPb(Br_0.4_I_0.6_)_3_ crystallites of up to 100 nm side length segregated into pure CsPbI_3_ and secondary phases observable by TEM. Furthermore, the TEM analyses give insights into the phase segregation on the sub-nm scale in mixed-halide perovskite crystallites of side lengths of 40 to 50 nm.

**Figure 9:**
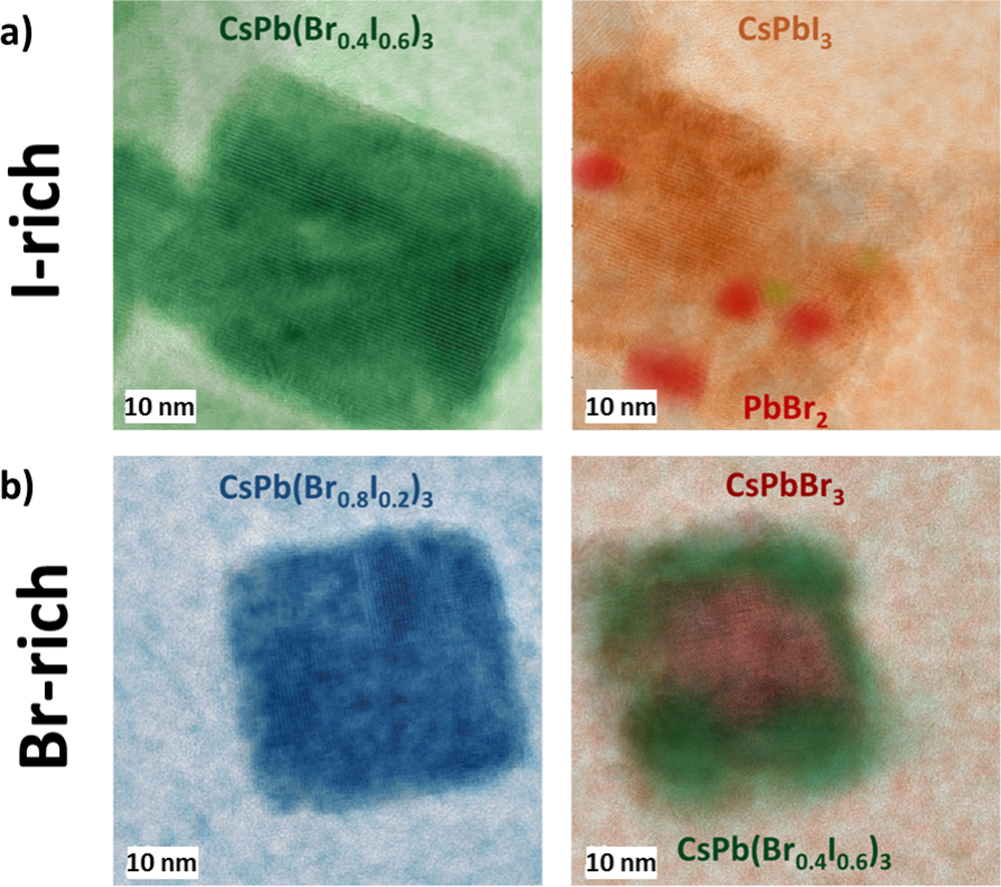
Halide segregation in (a) I-rich and (b) Br-rich crystallites. While the I-rich initial crystallite segregates into CsPbI_3_ with Br-rich inclusions, the Br-rich initial crystallite segregates into CsPbBr_3_ a core and an iodide-richer phase at the edges.

Figure 9 shows the phase segregation in an iodide-rich crystallite of the present work in comparison with the bromide-rich crystallites from Funk et al.^50^ In Figure 9b, a clear direction is observable for the phase segregation in the Br-rich CsPb(Br_0.8_I_0.2_)_3_ crystallite. An I-rich phase segregates at the edges of the particle, while a pure CsPbBr_3_ domain forms at the center. In contrast, the I-rich CsPb(Br_0.4_I_0.6_)_3_ sample (Figure 9a) segregates into a pure CsPbI_3_ matrix with small Br-rich inclusions (CsPbBr_3_ or PbBr_2_). The spatial distribution of both phases in the segregated condition is characteristic for the initial ratio. However, both initially bromide-rich and iodide-rich ratios have in common that the main phase forms according to the halide with the larger concentration in the compound. To provide an explanation for this behavior, the following pathway is proposed, which at the same time explains why electron-beam and light irradiation can both trigger phase segregation in LHP compounds.

### 4.1 Initiating and Driving Halide Segregation: Interplay of Halide Oxidation, Band Gap Differences, and Lattice Strain

For halide perovskites, radiolysis has been established as the main damaging process when irradiated by an electron beam. Radiolysis can be rationalized as the interaction of the materials electrons with the electromagnetic field of the incoming electrons and causes the oxidation of the halide anion as well as the reduction of the lead cation.^37^ As described in the Introduction, the same oxidation process has recently emerged in the halide perovskite community to be the initial step of halide phase segregation induced by light irradiation or by applied voltage. The present work provides further confirmation that halide oxidation indeed plays a key role in halide segregation.

**Figure 10:**
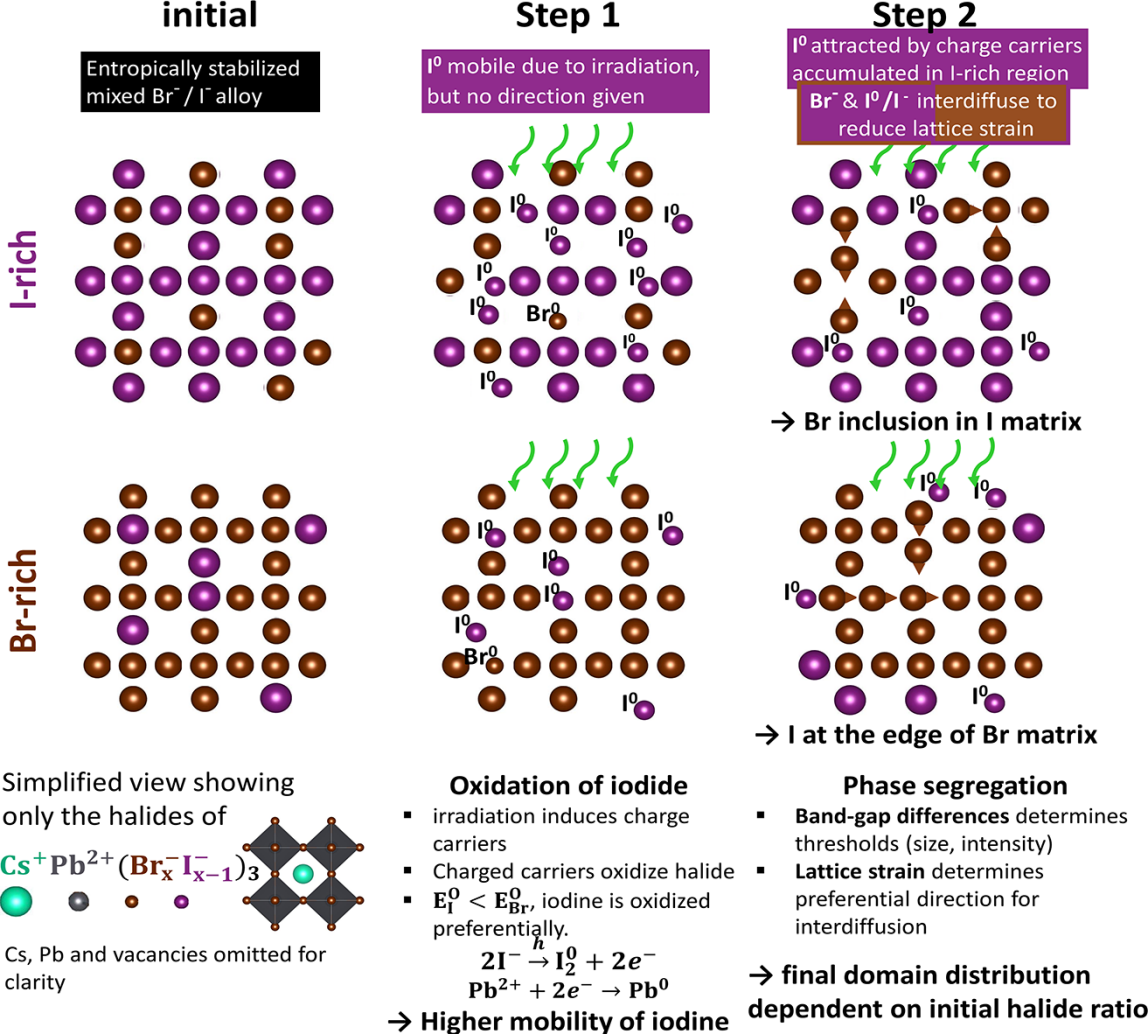
Schematics of proposed mechanism for halide phase segregation in initially I-rich and Br-rich LHP.

The schematics in Figure 10 show a simplification of the segregation process step by step. In short, first, the iodide (purple) is oxidized by the incoming irradiation, leading to a higher iodine mobility. The band gap differences and lattice strain give the condition for phase segregation and preferential direction for the fast moving iodine, while the bromide (brown) follows via slower processes to satisfy the stoichiometry and release of lattice strain.

The simplified view in Figure 10 shows only the halides of the initially I-rich (upper row) and Br-rich (lower row) mixed-halides. The initial mixed state under equilibrium conditions is depicted at the left.

Step 1: The incoming electromagnetic field (electron beam or light) excites an electron from the valence-band to the conduction-band.^38^ The valence-band maximum consists of a halide orbital.^70^ The induced hole densities lead to the oxidation of the halide anion and subsequently result in the breaking of the X–Pb bond. Because the oxidation potential E^O^ is higher for Br than for I, I is oxidized preferentially. The oxidation potential E^O^ for the formation of molecular iodide or bromide via 2X^–^ → 2X_2_^0^ + 2e^–^, is *E*_I_^O^ = + 0.535 V and *E*_Br_^O^ = + 1.065 V.^22,24,71^ Therefore, chemically, the oxidation of bromide is not relevant, as long as there is iodide to oxidize. This also rationalizes the reported expulsion of iodide,^22−24^ even though kinetically, Br^0^, Br^*–*^, or Br_2_ should all be more mobile due to their smaller radius than I^0^, I^*–*^, or I_2_.^72,73^ The oxidation of iodide is a reversible redox reaction (oxidation: 2I^–^ → 2I + 2e^–^; reduction: Pb^2+^ + 2e^–^ → Pb^0^; redox reaction: 2I^–^ + Pb^2+^ → 2I + 2Pb^0^). However, the reversibility is dependent on the spatial distribution of the oxidized products after the initial process.^24^

The oxidation is the fundamental origin of the halide phase segregation and explains why the iodine is more mobile and moves further. Due to this oxidation process it is rather iodide, than bromide that outgases for NPs smaller than 23 nm, causing the blue shift of the PL peak. However, iodide oxidation alone cannot rationalize the preferential direction that is necessary for segregation to occur. Furthermore, the present work shows that not only the oxidized iodine but also the Br diffuses in NPs with sizes *>*40 nm. Iodine (whether in its radical I^0^ or molecular I_2_ form cannot be told from the present results) may move interstitially mediated^22^ or diffuse via vacancies.^26,73^ Vacancies can be assumed to be present from the beginning. The faster diffusion of iodide in comparison to bromide causes the occurrence of a Kirkendall effect, leading to the formation of further vacancies. This situation facilitates the interdiffusion via vacancy-mediated processes following the movement of the iodine to satisfy the stoichiometry.

Step 2: As a condition for halide phase segregation, the threshold values for excitation intensity and particle size also observed in the present work have to be surpassed. This can be explained by the band gap-based model, which is dependent on the excitation intensity *I*_exc_ as well as on the carrier diffusion length *L*_e/h_*.*^9^ The differences in band gap energies between I-rich and Br-rich regions cause charge-carrier accumulation in I-rich domains, which again causes the mobile iodine to diffuse to these regions.

To explain the preferential direction in dependence of the initial halide ratio, depicted in Figure 10, the following mechanism is proposed. While the I-rich compound turns into a CsPbI_3_ matrix with small Br-rich inclusions, for the Br-rich sample, a CsPbBr_3_ domain forms in the center, while iodine segregates to the edges. The common element of the spatial evolution starting from the two different initial [Br]:[I] ratios in the crystallites with sizes of *>*40 nm is that the material systems tend to build up the pure CsPbX_3_ phase of the predominant halide X. This supports our proposal that the following mechanism introduced in Funk et al.^50^ drives the segregation: The difference in Shannon ionic radii for Br and I^74^ result in an asymmetry in bonding strengths (see Table S2) and causes the lattice to be strained. The spatial evolution of the phases strongly suggests that, under the condition that the bromide is less mobile than the iodide, the systems seek strain-release by evolving into the pure phase of the predominant phase.

The interplay of three factors, iodine oxidation, differences in band gap energy and lattice strain, can rationalize the various results obtained in the present work. Furthermore, it explains why electron-beam and light irradiation can both trigger phase segregation in LHP compounds.

## 5 Conclusions

The present work investigated halide phase-segregation in nanocrystals of mixed-halide CsPb(Br_*x*_I_1*–x*_)_3_ in dependence on their sizes and their initial halide [Br]:[I] ratios. The segregation was induced and monitored in situ by HRTEM on the sub-nm level, and the results were correlated with light-induced phase segregation monitored by PL spectroscopy. From in situ HRTEM measurements of inorganic LHP NPs, a clear direction for electron-beam-induced phase segregation was observable in a Br-rich crystallite, where an I-rich phase segregated at the borders of the particle while a CsPbBr_3_ domain formed at the center. In contrast, the I-rich crystallite becomes a CsPbI_3_ matrix with small, Br-rich inclusions. PL analyses using light irradiation on the identical sample indicated the same phase segregation to pure CsPbI_3_. No phase segregation was observed by HRTEM or PL in particles of side length of *≤*22 nm, confirming an NP size threshold below which no phase segregation takes place. However, the PL peaks acquired on NPs with side lengths of *≤*22 nm exhibited a blue-shift of about 3 nm, indicating the loss of iodine.

The presented results show that to rationalize halide phase segregation, the interaction of three partial processes has to be considered. (1) Irradiation-induced iodide oxidation results in higher mobility of iodine than bromide. In combination with this process, (2) intrinsic differences in band gap energy explain the size and intensity thresholds below which no phase segregation but instead iodine expulsion occurs, and (3) intrinsic lattice strain provides the preferential direction in dependence of the initial halide ratio. Furthermore, since the oxidation can be induced by both electron-beam and light irradiation, both radiation sources can trigger phase segregation in LHP compounds.

## Supporting Information Available

Literature overview of PL peaks for different halide ratios in CsPb(Br_*x*_I_1–*x*_)_3_; calculated bonding lengths for CsPbBr_3_ and CsPbI_3_; interplanar spacings for CsPbBr_3_, CsPbI_3_; PbBr_2_, PbI_2_, and Pb; further absorption and PL measurements; additional HRTEM time series with corresponding MA abundance maps; HRTEMs with more detailed phase assignment; details on the influence of residual organic material including HRTEM and low-magnification TEM images; detailed analysis of heat effects in the electron beam (PDF)
