# Supplementary material for: Phase Segregation Mechanisms in Mixed-Halide CsPb(BrxI1–x)3 Nanocrystals in Dependence of Their Sizes and Their Initial [Br]:[I] Ratios
Source: ACS Mater Au. 2023 Sep 6;3(6):687–98. doi: 10.1021/acsmaterialsau.3c00056 (PMC10636778; doi:10.1021/acsmaterialsau.3c00056)
Supplement: Supplementary file 1 — mg3c00056_si_001.pdf [file mg3c00056_si_001.pdf]

Phase segregation mechanisms in mixed-halide $CsPb(Br_xI_{1-x})_3$ nanocrystals in dependence of their sizes and their initial [Br]:[I] ratios

*Hannah Funk[1], Tal Binyamin[2], Lioz Etgar[2], Oleksandra Shargaieva[1], Thomas Unold[1], Alberto Eljarrat[3], Christoph T. Koch[3], Daniel Abou-Ras[1]**

[1] Helmholtz-Zentrum Berlin für Materialien und Energie GmbH,

[2] The Hebrew University of Jerusalem, Jerusalem, Israel

[3] Humboldt-Universität zu Berlin, Institut für Physik, Newtonstraße 15, 12489 Berlin, Germany

* daniel.abou-ras@helmholtz-berlin.de

1. Supplementary information on inorganic mixed halides

Table S1: PL peak λ in nm for different halide ratios in $CsPb(Br_xI_{1-x})_3$ and different samples with different particle size. $*$ ensemble film measured $**$ single NP measure.

Br/I ratio / sample	100/0	80/20	67/33	60/40	50/50	40/60	33/67	20/80	0/100
Bulk[1]	520	560		605		640	650	680	690
NP (9.6 nm)[2]	540		580	590	605	620/30	660		690
NP (11.8nm)[3]	510								
NP(11.8-14.3nm)[4]	510	530			590			670	690
NP(23nm)[5] $*$						635			690
NP(23nm)[5] $**$						615			690
NPlatelets (9-29μm)[6]	527								700
NP (10nm)[7]									700
NP (10nm)[8]	520								700
NP (10nm)[9]	505								

Table S2: Bonding lengths and type calculated with Program Jana2006[10] for lattice parameters 5.874 Å for $CsPbBr_3$[11] and 6.289 Å for $CsPbI_3$.[12]

Bonding type	Bonding length [Å] [Program Jana2006]	Shannon radius [Å]		
Cs-Br	4.15	3.63	-0.51	weak bonding
Pb-Br	2.94	3.15	+0.21	covalent
Cs-I	4.45	3.87	-0.58	weak bonding
Pb-I	3.15	3.39	+0.24	covalent

Table S3: Interplanar spacing for lattice planes hkl in Å for cubic $CsPbBr_3$[11] and $CsPbI_3$[12]

[Å]	$CsPbBr_3$	$CsPbI_3$	40:60
100	5.874	6.2894	6.1232
110	4.1535	4.4472	4.3297
111	3.3913	3.6311	3.5351
200	2.937	3.1447	3.0616
210	2.6269	2.8127	2.7383
211	2.398	2.5676	2.4997
220	2.0767	2.2236	2.1648
300	1.958	2.0964	2.0410

Table S4: Interplanar spacing for lattice planes hkl in Å for $PbBr_2$ (space group no. 62)[13] and PbI_2 (space group no. 164)[14]

$PbBr_2$			PbI_2		
Relative intensity (%)	hkl	interplanar spacing [Å]	%	hkl	[Å]
17	002	4.759	40	001	6.99
23	011	4.226	5	100	3.948
52	102	4.095	100	011	3.437
31	200	4.019	29	102	2.617
71	111	3.7402	36	110	2.279
35	112	3.092			
23	011	3.071			
27	011	3.059			
12	011	2.951			
100	211	2.91244			
78	013	2.632			

Table S5: Interplanar distances for lattice planes hkl in Å for Pb Crystallography Open Database (COD) website. COD ID 9008477 : Pb is Fm-3m space group with cubic structure, cell parameters: *a* 4.950Å.

I	hkl	d
100	111	2.85
50	200	2.47
33	220	1.75
37	311	1.49
10	222	1.42

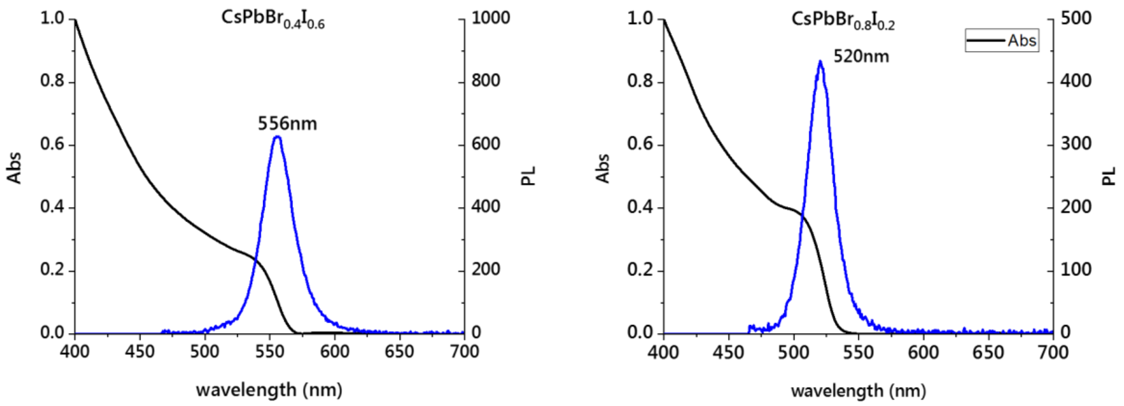

Figure S1: Absorption and PL of iodide rich $CsPb(Br_{0.4}I_{0.6})_3$ NP and bromide richt$CsPb(Br_{0.8}I_{0.2})_3$ NP, displaying a band gap energy of 556 nm and 520 nm respectively. Measurement performed by Tal Binymain, HUJ Israel.

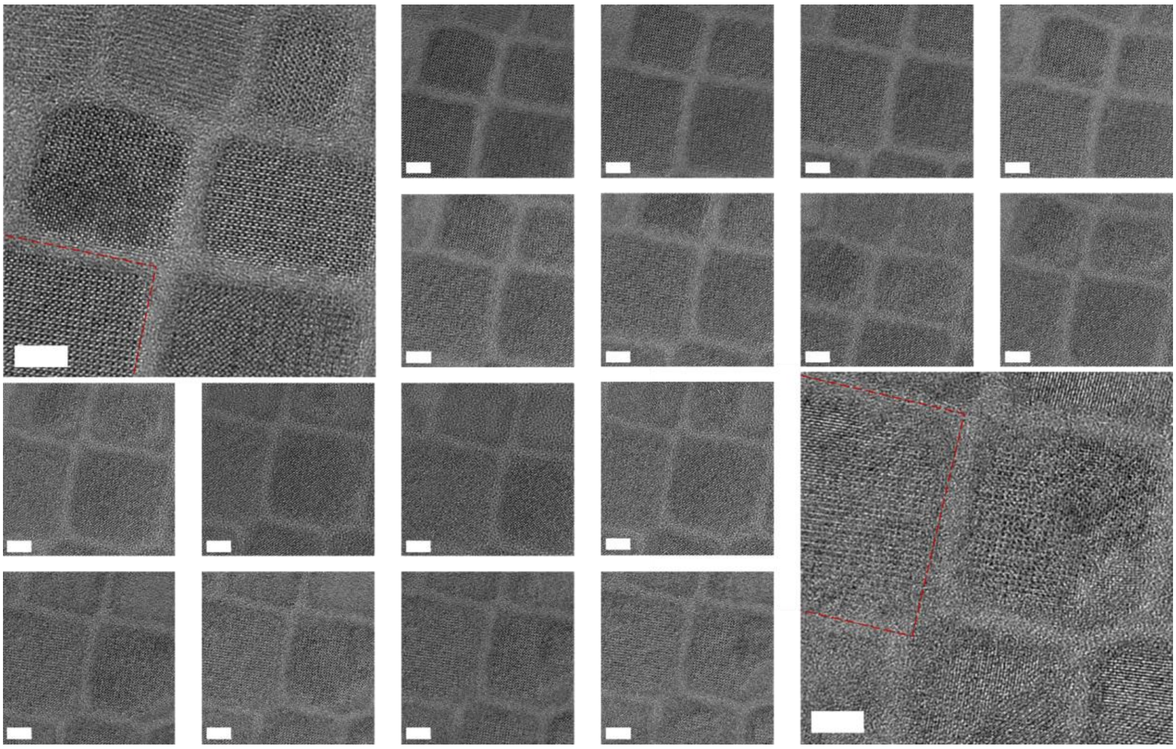

Figure S2: HRTEM time series of 17 min (each image 1 min) of $CsPb(Br_{0.8}I_{0.2})_3$ NP showing no halide phase segregation, but a slow amorphization. Sale bar 5nm.

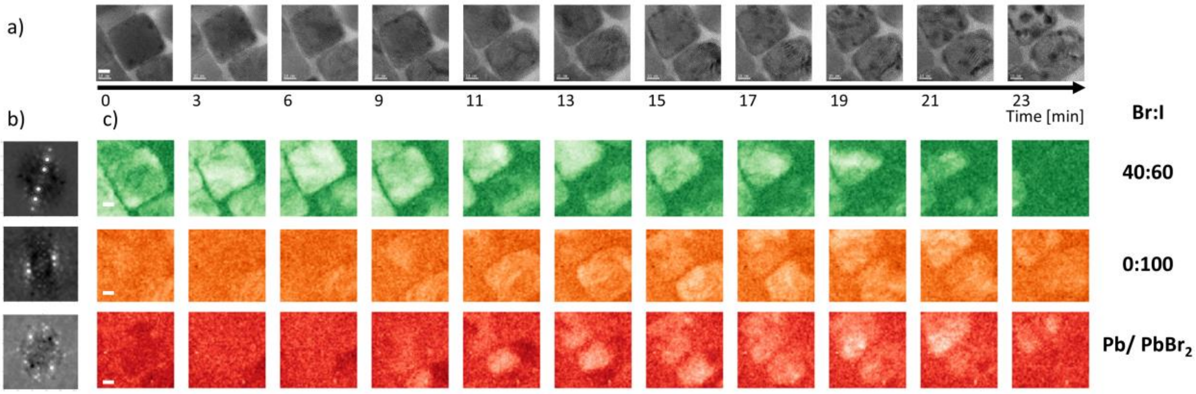

Figure S3: Time series of initial $CsPb(Br_{0.4}I_{0.6})_3$ **Position B**: 40nm x 40nm upper crystallite segregating a) HRTEM images acquired during 23 min of total acquisition duration b) characteristic diffraction patterns of the three abundant structures identified by MA c) corresponding abundance maps identified as $CsPb(Br_{0.4}I_{0.6})_3$ segregating into $CsPbI_3$ and Pb or $PbBr_2$. High intensity in a pixel corresponds with a high abundance of the structure in this pixel. Scale bars 10nm.

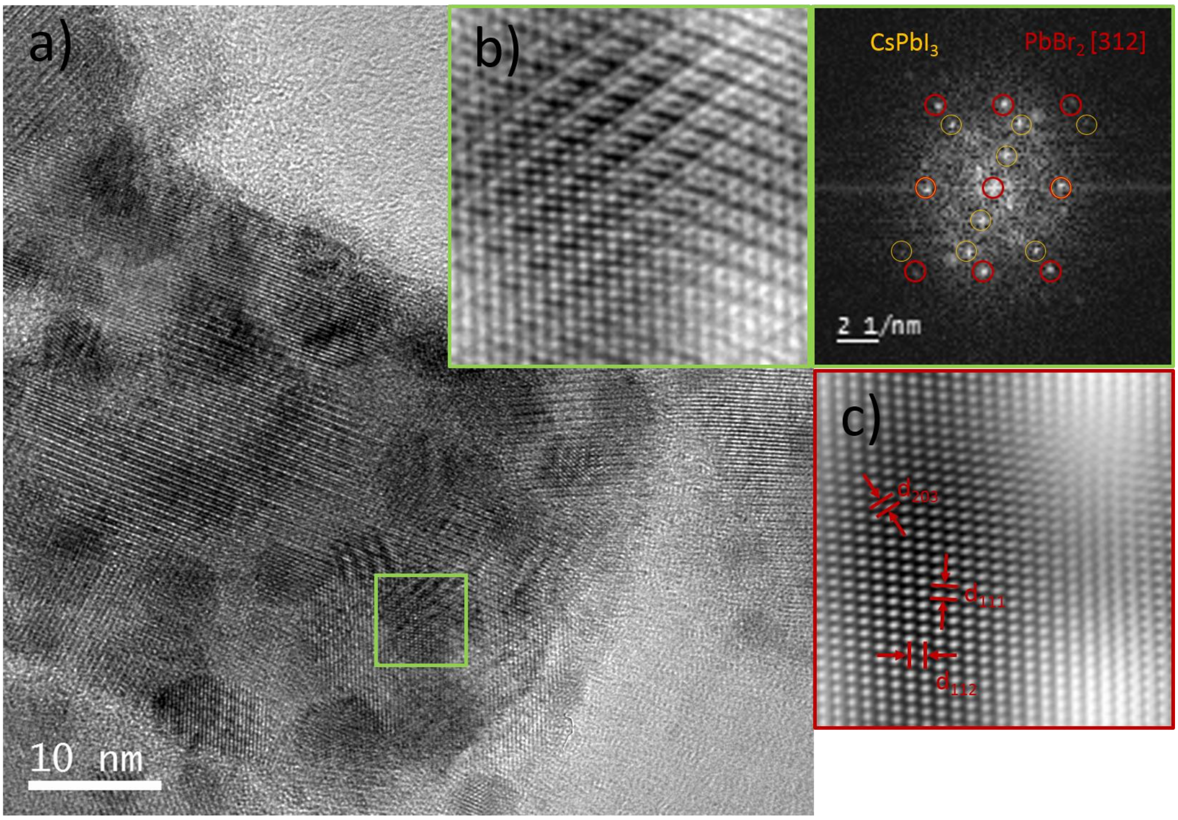

Figure S4: Assignment of phases for small inclusion: a) HRTEM at position B after 10 min of electron beam irradiation. b) Detail from image and its FFT, displaying the ED pattern of $CsPbI_3$ and $PbBr_2$ in viewing direction [312] c) Fourier filtered image was used for more exact measurement of the interplanar distances.

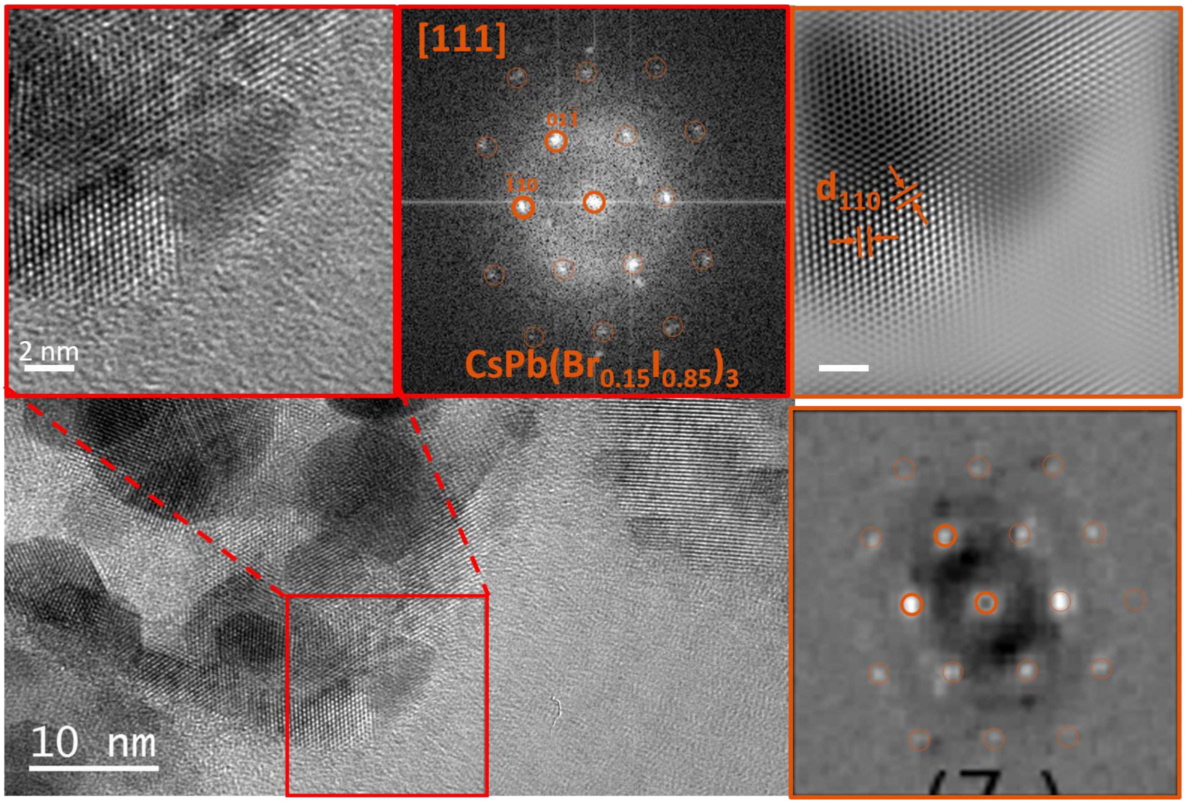

Figure S5: Assignment of phases at position B. HRTEM after 13 min of electron beam irradiation. For phase assignment the FFT of the image detail (red border) matching the diffraction pattern determined by the algorithm (orange border) is chosen, displaying the ED pattern of $CsPb(Br_{0.15}I_{0.85})_3$ in viewing direction [111]. The exact interplanar distances are obtained from the Fourier filtered image

2. Residual organic material

It is noteworthy that the time scales of the processes in the time series described above differ substantially. While the halide phase segregation at position A (Figure 3.6) occurred during the first 12 min, at position B, the phase segregation occurred already during the first 5 min (Figure 3.8). This means that more than twice the total electron dose was necessary to induce phase segregation at position A, in comparison with the particle at position B.

Other particles did not show any phase decomposition during expositions of up to 20 min, as shown in Figures S6a-c, where no substantial change can be observed in the HRTEM images or in their FFTs at position C. Again at other positions, the decomposition was so fast under the identical conditions as at position C, that halide phase segregation was not possible to analyzed before the particle decomposed (see position D in Figures S6d-f). The overview images in Figures S6g-i show the substantial difference between the time evolution of the particles at positions C and D. While the NPs at position C in Figure S6g remain intact even after 20 min of electron irradiation, the NPs at position D in Figures S6h and i, before and after 7 min of electron-beam irradiation, are completely decomposed. Furthermore, the overview images show that this result is not related to the shape of the NPs or to other properties of the corresponding crystallite (as e.g., attached ligands), but that different regions of the sample behave differently under the electron beam.

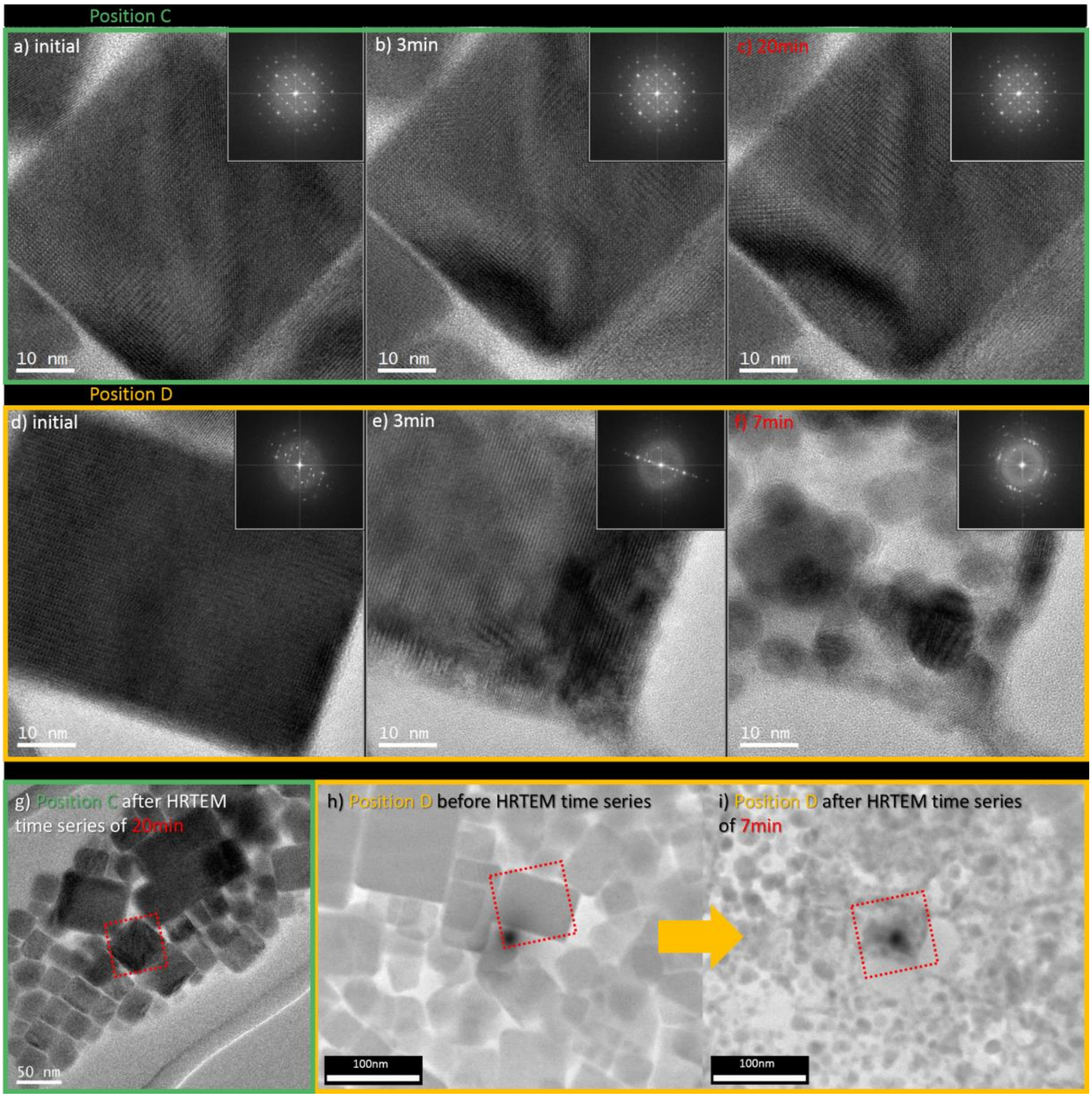

Figure S6: HRTEM images of time series of $CsPb(Br_{0.4}I_{0.6})_3$ crystallites from two different positions (C and D) with identical imaging conditions, showing no (position C) and very fast decomposition (position D). a)-c) Position C does not show loss of crystallinity or transformation over 20 min of electron-beam irradiation. d-f) Position D decomposes thoroughly during 7 min of irradiation. g) Overview image of position C showing an intact environment after 20 min irradiation. h) showing position D before and i) after the time series.

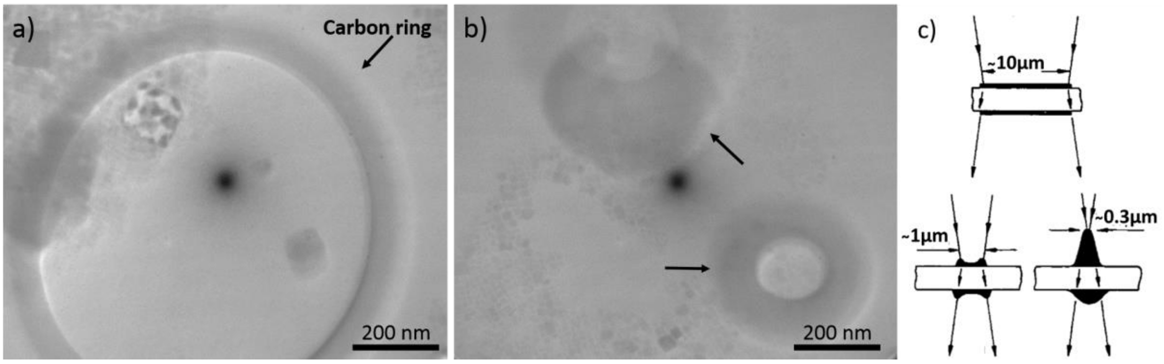

Figure S7: Low-magnification TEM images showing three examples of visible contamination due to the electron beam after several minutes irradiation: a) carbon ring for a large condenser aperture as used in the HRTEM time series, resulting in a illumination area of about $d = 1$ µm and b) carbon rings for a smaller aperture, illuminating an area of about $d = 0.4$ µm exhibiting contamination in accordance with the c) schematic illustration of the form in which the carbon contaminant growth with decreasing electron beam size. Adapted from Hren.[15]

This different behavior in the electron beam is likely caused by local fluctuations of residual organic material from the solution in which the NP were dispersed and drop-cast. Further organic content stems from the ligands originally attached to the NPs surface itself. Figure S7a and b show overview images of positions where HRTEM time series were acquired. The distinct "carbon rings" that were not present before the time series, indicate a strong contamination of the sample with organic material. The process of the decomposition and deposition of hydrocarbonates by the electron-beam as well as the subsequent diffusion of adsorbates into the illuminated area from the surrounding area is a complex process influenced by many parameters and the the physical details of this process cannot be determined by TEM[15,16] and therefore, only its effect is discussed here. Figure S7c shows a schematic illustration of the form, in which carbon contamination growth in dependence of the electron-beam diameter. While large beam diameters cause a thin even growth, smaller diameters as used for HRTEM of about 1 µm result in the growth of elevations at the border of the illuminated area, while for very small beam diameters as used for STEM pillow growth is expected. The visible contamination in the overview image in Figures S7a and b for the larger (about 1 µm) and smaller (about 0.4 µm) beam diameter, hence, corresponds to the expected profile for contamination growth. During the time series the deposited carbon layer acts as a protective layer since there is scattering of the incoming electrons[16] that is not happening in the sample itself, but in the carbon layer. The key point of its mitigating phase transformations in the electron beam is, however, that it is hindering the escape of halide ions from the sample. The suppression of degradation by carbon coating was also reported by Chen et al. for $MAPI_3$ in TEM and SEM measurements and attribute to the impediment of ion loss by the coating.[17] These effects vary with the thickness of the carbon layer and hence, with the available organic material in the environment of the electron beam illuminated area.

The contamination during the time series acquired from the spin-coated sample, as described in Funke et al.[18], was marginal. No contamination comparable to the "carbon rings" of the NP samples were visible. These differences stem from the different synthesis and preparation processes. Probably, the main factor is that the spin-coated samples are heated after deposition, a step in which the residual organic material dissociates. In contrast, the NPs are stored in a solution that, even if it is not the solution in which the reaction took place and "washed", will sill contain traces of residual material. The electron beam dosage causing phase segregation were about 4 times larger for the NP samples in the present work in comparison to the electron

dose rate used to induce the phase segregation in the spin-coated sample in Funke et al.[18] (see Table S6 for exact values). Due to the fact that the same electron beam conditions at different positions of the very same sample had quite different transformation effects in the NPs (see Figure S6), it is clear that the residual material plays a role in how fast and if the phase segregation takes place. Additionally, it may be that the NPs exhibit fewer defects and therefore, are more stable in comparison with the spin-coated sample, or that the ligands have an influence on the stability in the electron beam. However, from the results presented above it cannot be concluded on what happens to the ligands under the electron beam and whether or not they really play a role in the stabilization apart from preventing Oswald ripening, i.e., growth of larger particles in solution.

3. Heat effects

Another important aspect when considering the electron-matter interaction in the TEM is beam heating.[19] The temperature rise is given by the following equation

$$\Delta T_{max} = i_P \frac{(dU_P/dz)/e}{2\pi\kappa}(1/2 + \ln s/b),$$

where b is the radius of irradiated area, s is the distance to copper grid (thermal anchor), κ is the thermal conductivity, $i_P = \pi b^2 j_P$ the total primary beam current with the electron current density j_P and (dU_P/dz) is the fraction of inelastic scattered electrons.[20] The temperature rise is proportional to the total primary beam current and plotted for various materials in Figure S8. In accordance with the available literature, a value of about 0.4 $Wm^{-1}K^{-1}$ was assumed for the thermal conductivity of $CsPbI_3$ and $CsPbBr_3$.[21,22]

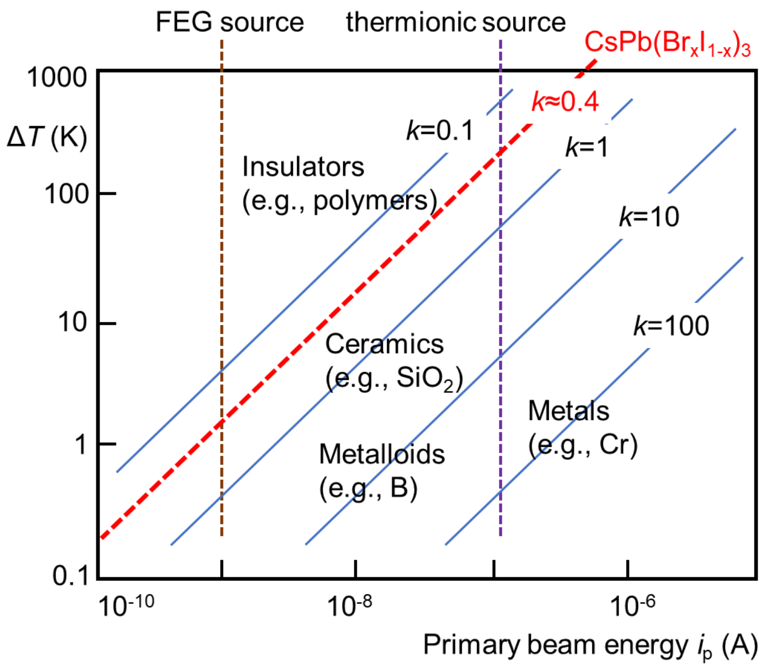

Figure S8: Temperature rise ΔT for various materials with different thermal conductivity κ in dependence of the primary beam current i_P. Figure redrawn from Ref. 23.

The primary beam current $i_P = \pi r^2 j_P$ for the HRTEM time series is calculated in Table S6.

Table S6: Electron dose and total beam current i_P for the HRTEM time series

	Dose [e/sÅ^2]	i_P [A] after 1 s	i_P [A] after 1 min	i_P [A] after 10 min
Funke et al.[18]	466	7×10^{-10}	4×10^{-8}	4×10^{-7}
Position A<br><br>(CL1 r=2×10^3 Å)	2246	2×10^{-8}	1.37×10^{-6}	1×10^{-5}
Position B<br><br>(CL1 r=2×10^3 Å)	1684	2×10^{-8}	1.03×10^{-6}	1×10^{-5}

While for the time series in Funke et al.[18], the primary beam current after 1 min corresponds to a heating of about 50 K, the primary beam current after 1 min for the measurements in the present work would exceed an increase of about 1000 K, in case the influence of the contamination layer is not considered. However, the thermal conductivity of amorphous carbon of up to 2.2 $Wm^{-1}K^{-1}$ should effectively diminish the temperature increase.[24–26]

For a [Br]:[I] ratio of 80:20 in an extended $CsPb(Br_xI_{1-x})_3$ crystal, the transformation from the γ to the β phase should take place at about 340 K and from the β to the α phase at 380 K. Assuming a room temperature of 22 °C, the cubic phase would be reached within the first 2 min. For a [Br]:[I] ratio of 40:60, the $\delta \rightarrow \alpha$ transformation takes place at 460 K. However, different from an extended $CsPb(Br_xI_{1-x})_3$ crystal, in iodine-rich NP, the orthorhombic γ phase is also present at room temperature. For NPs, temperature-dependent phase analyses are difficult to conduct owing to ligand desorption and the subsequent Oswald ripening. Oswald ripening resulting in the growth of larger particles (i.e., bulk material) starts to occur slowly from about 100 °C and fast for temperatures higher than about 225 °C.[9] However, for NPs dispersed on a TEM grid, the temperature related phase transformations should not be much of a concern since the coordination of the octahedra in $CsPb(Br_xI_{1-x})_3$ NP is the same for all temperature-related phase transformations. Moreover, heating leads to a higher symmetry (orthoromibic -> tetragonal -> cubic) realized by a compression of the *c* axis and a tilting of the octahedra which should be feasible to happen on the grid even if the ligands dissociate. For extnded $CsPbBr_3$ crystals, an onset temperature of about 580 °C (853 K) is reported for evaporation/decomposition.[27] Hence, it would be better to use TEM facilities with sample cooling capability to avoid high temperatures that definitely accelerates transformation processes.[28,29]

REFERENCES

(1) Beal, R. E.; Slotcavage, D. J.; Leijtens, T.; Bowring, A. R.; Belisle, R. A.; Nguyen, W. H.; Burkhard, G. F.; Hoke, E. T.; McGehee, M. D. Cesium Lead Halide Perovskites with Improved Stability for Tandem Solar Cells. *J. Phys. Chem. Lett.* **2016**, *7* (5), 746–751. https://doi.org/10.1021/acs.jpclett.6b00002.

(2) Chen, Q.; Wu, J.; Ou, X.; Huang, B.; Almutlaq, J. M.; Zhumekenov, A. A.; Guan, X.; Han, S.; Liang, L.; Yi, Z.; Li, J.; Xie, X.; Wang, Y.; Li, Y.; Fan, D.; Teh, D. B. L.; All, A. H.; Mohammed, O. F.; Bakr, O. M.; Wu, T.; Bettinelli, M.; Yang, H.; Huang, W.; Liu, X. All-Inorganic Perovskite Nanocrystal Scintillators. *Nature* **2018**, *561* (7721), 88–93. https://doi.org/10.1038/s41586-018-0451-1.

(3) Protesescu, L.; Yakunin, S.; Bodnarchuk, M. I.; Krieg, F.; Caputo, R.; Hendon, C. H.; Yang, R. X.; Walsh, A.; Kovalenko, M. V. Nanocrystals of Cesium Lead Halide Perovskites (CsPbX3, X = Cl, Br, and I): Novel Optoelectronic Materials Showing Bright Emission with Wide Color Gamut. *Nano Lett.* **2015**, *15* (6), 3692–3696. https://doi.org/10.1021/nl5048779.

(4) Gualdrón-Reyes, A. F.; Yoon, S. J.; Barea, E. M.; Agouram, S.; Muñoz-Sanjosé, V.; Meléndez, Á. M.; Niño-Gómez, M. E.; Mora-Seró, I. Controlling the Phase Segregation in Mixed Halide Perovskites through Nanocrystal Size. *ACS Energy Lett.* **2019**, *4* (1), 54–62. https://doi.org/10.1021/acsenergylett.8b02207.

(5) Zhang, H.; Fu, X.; Tang, Y.; Wang, H.; Zhang, C.; Yu, W. W.; Wang, X.; Zhang, Y.; Xiao, M. Phase Segregation Due to Ion Migration in All-Inorganic Mixed-Halide Perovskite Nanocrystals. *Nat. Commun.* **2019**, *10* (1), 1–8. https://doi.org/10.1038/s41467-019-09047-7.

(6) Zhang, Q.; Su, R.; Liu, X.; Xing, J.; Sum, T. C.; Xiong, Q. High-Quality Whispering-Gallery-Mode Lasing from Cesium Lead Halide Perovskite Nanoplatelets. *Adv. Funct. Mater.* **2016**, *26* (34), 6238–6245. https://doi.org/10.1002/adfm.201601690.

(7) Bi, C.; Kershaw, S. V.; Rogach, A. L.; Tian, J. Improved Stability and Photodetector Performance of CsPbI3 Perovskite Quantum Dots by Ligand Exchange with Aminoethanethiol. *Adv. Funct. Mater.* **2019**, *29* (29), 1–9. https://doi.org/10.1002/adfm.201902446.

(8) Nedelcu, G.; Protesescu, L.; Yakunin, S.; Bodnarchuk, M. I.; Grotevent, M. J.; Kovalenko, M. V. Fast Anion-Exchange in Highly Luminescent Nanocrystals of Cesium Lead Halide Perovskites (CsPbX3, X = Cl, Br, I). *Nano Lett.* **2015**, *15* (8), 5635–5640. https://doi.org/10.1021/acs.nanolett.5b02404.

(9) Scheidt, R. A.; Atwell, C.; Kamat, P. V. Tracking Transformative Transitions: From CsPbBr3 Nanocrystals to Bulk Perovskite Films. *ACS Mater. Lett.* **2019**, *1* (1), 8–13. https://doi.org/10.1021/acsmaterialslett.9b00001.

(10) Petrícek, V.; Dušek, M.; Palatinus, L. Crystallographic Computing System JANA2006: General Features. *Zeitschrift fur Kristallographie*. 2014, pp 345–352. https://doi.org/10.1515/zkri-2014-1737.

(11) Moller, C. K. Crystal Structure and Photoconductivity of Caesium Plumbohalides. *Nature* **1958**, *182*, 1436. https://doi.org/10.1038/1821436a0.

(12) Trots, D. M.; Myagkota, S. V. High-Temperature Structural Evolution of Caesium and Rubidium Triiodoplumbates. *J. Phys. Chem. Solids* **2008**, *69* (10), 2520–2526. https://doi.org/10.1016/j.jpcs.2008.05.007.

(13) PbBr2 Rt Crystal Structure: Datasheet from ``PAULING FILE Multinaries Edition -- 2012''. *Springer Mater.* **2012**.

(14) PbI2 Rt 2H Crystal Structure: Datasheet from ``PAULING FILE Multinaries Edition -- 2012''. *Springer Mater.* **2012**.

(15) Hren, J. J. Specimen Contamination in Analytical Electron Microscopy: Sources and Solutions. *Ultramicroscopy* **1979**, *2* (9), 375–380.

(16) Hettler, S.; Dries, M.; Hermann, P.; Obermair, M.; Gerthsen, D.; Malac, M. Carbon Contamination in Scanning Transmission Electron Microscopy and Its Impact on Phase-Plate Applications. *Micron* **2017**, *96* (February), 38–47. https://doi.org/10.1016/j.micron.2017.02.002.

(17) Chen, S.; Zhang, Y.; Zhang, X.; Zhao, J.; Zhao, Z.; Su, X.; Hua, Z.; Zhang, J.; Cao, J.; Feng, J.; Wang, X.; Li, X.; Qi, J.; Li, J.; Gao, P. General Decomposition Pathway of Organic–Inorganic Hybrid Perovskites through an Intermediate Superstructure and Its Suppression Mechanism. *Adv. Mater.* **2020**, *32* (29), 1–7. https://doi.org/10.1002/adma.202001107.

(18) Funk, H.; Shargaieva, O.; Eljarrat, A.; Unger, E. L.; Koch, C. T.; Abou-Ras, D. In-Situ TEM Monitoring of Phase-Segregation in Inorganic Mixed Halide Perovskite. *J. Phys. Chem. Lett.* **2020**, *11*, 4945–4950. https://doi.org/10.1021/acs.jpclett.0c01296.

(19) Egerton, R. F. Radiation Damage to Organic and Inorganic Specimens in the TEM. *Micron* **2019**, *119* (January), 72–87. https://doi.org/10.1016/j.micron.2019.01.005.

(20) Hobbs, L. W. Radiation Effects in Analysis of Inorganic Specimens by TEM. In *Introduction to Analytical Electron Microscopy*; Hren, J. J., Goldstein, J. I., Joy, D. C., Eds.; Springer US: Boston, MA, 1979; pp 437–480. https://doi.org/10.1007/978-1-4757-5581-7_17.

(21) Lee, W.; Li, H.; Wong, A. B.; Zhang, D.; Lai, M.; Yu, Y.; Kong, Q.; Lin, E.; Urban, J. J.; Grossman, J. C.; Yang, P. Ultralow Thermal Conductivity in All-Inorganic Halide Perovskites. *Proc. Natl. Acad. Sci.* **2017**, *114* (33), 201711744. https://doi.org/10.1073/pnas.1711744114.

(22) Haeger, T.; Wilmes, M.; Heiderhoff, R.; Riedl, T. Simultaneous Mapping of Thermal Conductivity, Thermal Diffusivity, and Volumetric Heat Capacity of Halide Perovskite Thin Films: A Novel Nanoscopic Thermal Measurement Technique. *J. Phys. Chem. Lett.* **2019**, *10* (11), 3019–3023. https://doi.org/10.1021/acs.jpclett.9b01053.

(23) Williams, D. B.; Carter, C. B. *Transmission Electron Microscopy: A Textbook for Materials Science*; 2009. https://doi.org/10.1007/978-0-387-76501-3.

(24) Ho, C. Y.; Powell, R. W.; Liley, P. E. *Thermal Conductivity of the Elements*; 1972; Vol. 1. https://doi.org/10.1063/1.3253100.

(25) Balandin, A. A. Thermal Properties of Graphene and Nanostructured Carbon Materials. *Nat. Mater.* **2011**, *10* (8), 569–581. https://doi.org/10.1038/nmat3064.

(26) Bullen, A. J.; O'Hara, K. E.; Cahill, D. G.; Monteiro, O.; Von Keudell, A. Thermal Conductivity of Amorphous Carbon Thin Films. *J. Appl. Phys.* **2000**, *88* (11), 6317–6320. https://doi.org/10.1063/1.1314301.

(27) Kulbak, M.; Gupta, S.; Kedem, N.; Levine, I.; Bendikov, T.; Hodes, G.; Cahen, D. Cesium Enhances Long-Term Stability of Lead Bromide Perovskite-Based Solar Cells. *J. Phys. Chem. Lett.* **2016**, *7* (1), 167–172. https://doi.org/10.1021/acs.jpclett.5b02597.

(28) Dang, Z.; Shamsi, J.; Palazon, F.; Imran, M.; Akkerman, Q. A.; Park, S.; Bertoni, G.; Prato, M.; Brescia, R.; Manna, L. In-Situ Transmission Electron Microscopy Study of Electron Beam-Induced Transformations in Colloidal Cesium Lead Halide Perovskite Nanocrystals. *ACS Nano* **2017**, *11* (2), 2124–2132. https://doi.org/10.1021/acsnano.6b08324.

(29) Dang, Z.; Luo, Y.; Xu, Y.; Gao, P.; Wang, X.-S. Transformation and Degradation of Metal Halide Perovskites Induced by Energetic Electrons and Their Practical Implications. *Nano Futur.* **2021**, *5* (3), 032001. https://doi.org/10.1088/2399-1984/ac0c24.
